# Combinatorial recognition of clustered RNA elements by the multidomain RNA-binding protein IMP3

**DOI:** 10.1038/s41467-019-09769-8

**Published:** 2019-05-22

**Authors:** Tim Schneider, Lee-Hsueh Hung, Masood Aziz, Anna Wilmen, Stephanie Thaum, Jacqueline Wagner, Robert Janowski, Simon Müller, Silke Schreiner, Peter Friedhoff, Stefan Hüttelmaier, Dierk Niessing, Michael Sattler, Andreas Schlundt, Albrecht Bindereif

**Affiliations:** 1 Institute of Biochemistry, Justus-Liebig-University of Giessen, 35392 Giessen, Germany; 2 Center for Integrated Protein Science Munich (CIPSM) at Department of Chemistry, Technical University of Munich (TUM), 85747 Garching, Germany; 3 Institute of Structural Biology, Helmholtz-Zentrum München, 85764 Neuherberg, Germany; 4 Faculty of Medicine, Institute of Molecular Medicine, Section for Molecular Cell Biology, Martin Luther University Halle-Wittenberg, 06120 Halle, Germany; 5 Institute of Pharmaceutical Biotechnology, Ulm University, 89081 Ulm, Germany; 6 Present Address: Institute for Molecular Biosciences and Center for Biomolecular Magnetic Resonance (BMRZ), Goethe-University Frankfurt, 60438 Frankfurt, Germany

**Keywords:** RNA metabolism, Structural biology

## Abstract

How multidomain RNA-binding proteins recognize their specific target sequences, based on a combinatorial code, represents a fundamental unsolved question and has not been studied systematically so far. Here we focus on a prototypical multidomain RNA-binding protein, IMP3 (also called IGF2BP3), which contains six RNA-binding domains (RBDs): four KH and two RRM domains. We establish an integrative systematic strategy, combining single-domain-resolved SELEX-seq, motif-spacing analyses, in vivo iCLIP, functional validation assays, and structural biology. This approach identifies the RNA-binding specificity and RNP topology of IMP3, involving all six RBDs and a cluster of up to five distinct and appropriately spaced CA-rich and GGC-core RNA elements, covering a >100 nucleotide-long target RNA region. Our generally applicable approach explains both specificity and flexibility of IMP3-RNA recognition, allows the prediction of IMP3 targets, and provides a paradigm for the function of multivalent interactions with multidomain RNA-binding proteins in gene regulation.

## Abstract (web summary)

Multidomain RNA-binding proteins recognize specific target sequences through mechanisms that are not well understood. Here the authors present an integrated approach to define the RNA-binding specificity and RNP topology and apply it to the analysis of the prototypical multidomain RNA-binding protein IMP3.

## Introduction

The insulin-like growth factor 2 mRNA-binding protein 3 (IMP3 or IGF2BP3) belongs to a family of three highly conserved RNA-binding proteins (IMP1, IMP2, and IMP3) that are involved in post-transcriptional gene regulation of mRNAs^1^. The three mammalian paralogs are often described as oncofetal due to their expression primarily during embryogenesis and severe phenotypes in the case of impaired expression^2,3^.

The currently best-understood IMP-mediated mechanism of modulating mRNA fate comprises the so-called safe housing of specific transcripts in mRNP granules^4^. This caging of mRNAs ranges in its functional spectrum from packaging for cytoplasmic transport^5^, delayed translation within stable mRNPs^6–8^, cytoplasmic storage, and protection against premature miRNA-directed mRNA regulation^3,9–12^. Several target mRNAs have been suggested^3,13^, with IMP1 associating with the *ACTB* mRNA zipcode element and all three IMPs regulating *HMGA2* stability via the 3′-UTR as the currently best-studied examples^9–12,14–16^.

In contrast to IMP1 and IMP2, the biological relevance of IMP3 has long been underestimated. Research on IMP3 largely focused on its association with many cancer-related tumor entities, since its re-expression correlates with a poor prognosis for patients, classifying IMP3 as a tumor marker^17–19^.

The IMP protein family represents a prototypical example of multidomain RBPs and is characterized by a common architecture of six potential RNA-binding units: two N-terminal RNA-recognition motifs (RRMs) and four consecutive hnRNP K-homology (KH) domains^1^. It has been a long-standing question how multiple RBDs cooperate in specific and high-affinity RNA-target recognition: Which of the individual domains are involved, what are their contributions, and how flexible is the RNA–protein interaction pattern?

Assessing the contributions and cooperativity of multiple RBDs in binding to multipartite RNA motifs is challenging, and a generally applicable approach has not been described so far. Due to the potential dynamic domain arrangements of multiple RBDs, structural studies require an integrated approach, combining solution techniques and crystallography^20–24^. For the IMPs, structural information is available only for single RRMs of IMP2 (RRM1, PDB-ID: 2CQH) and IMP3 (RRM2, PDB-ID: 2E44, both unpublished). The presence of a very short linker sequence suggests that the two domains are arranged in a compact tandem, which might drive their RNA specificity. Analogously, there is evidence that the KH1–2 and KH3–4 tandem domains represent prearranged RNA-binding modules for recognition of bipartite RNA sequence motifs. Structures of the human IMP1 KH3–4^14^, as well as the KH3–4 di-domains of the chicken ortholog ZBP1^16^ proved the existence of an extended domain interface between KH3 and 4. These structures suggest target RNA motifs to require a minimal spacing to be recognized by the tandem RBDs. For example, KH3–4 of ZBP1/IMP1 recognizes a combination of two sequence elements: CGGAC-N_10–25_-(C/A–CA–C/U) in both possible arrangements^14–16^.

Previous studies proposed short recognition sequences of IMPs, based on in vivo CLIP^3,13,25^ and in vitro selections (SELEX, RNAcompete, and Bind-N-seq)^5,26–28^, all suggesting an overall CA-rich consensus. However, the major limitation of in vitro selection approaches is that they usually start with short degenerate sequences, which can accommodate only a single RNA-binding motif. Therefore, the contributions of individual domains have remained elusive. Finally, while previous studies provide evidence for an essential role for KH domains in RNA interaction, no function had been ascribed yet to the two RRMs^5,14–16,29,30^.

To study IMP3 as a prototypical example of a multidomain RBP, we established a systematic, domain-resolved SELEX procedure coupled with RNA-seq and combinatorial bioinformatic approaches. Importantly, we used a very long degenerate sequence (N_40_) as a basis for SELEX, to allow multiple RNA contacts with more than a single RNA-binding domain, and a corresponding bioinformatic spacing analysis. This led us to the discovery that IMP3 recognizes—through the activity of all of its tandem RNA-binding domains—an extended array of multiple *cis*-acting RNA elements, composed of CA-rich motifs and sequences with a common GGC core. These biochemical findings are supported by integrated structural biology, combining crystallography and NMR for structural analysis and RNA-binding studies of IMP3 KH and RRM-tandem domains.

Taken together, we provide biochemical, bioinformatic, and structural evidence for recognition of an ordered array of RNA elements by IMP3, arranged in a certain spacing pattern and covering regions that can span more than 100 nts. This model is supported by the analysis of endogenous IMP3 target mRNAs, including the well-studied *HMGA2* transcript, for which we investigated the functional cross-regulation between IMP3 and the let-7 miRNA. In sum, we provide a framework for investigating large regulatory mRNP complexes. Thereby, we establish a general approach to systematically dissect complex and combinatorial RNP networks, which can be applied to any multidomain RNA-binding protein.

## Results

### IMP3 recognizes an array of distinct sequence elements

**Fig. 1:**
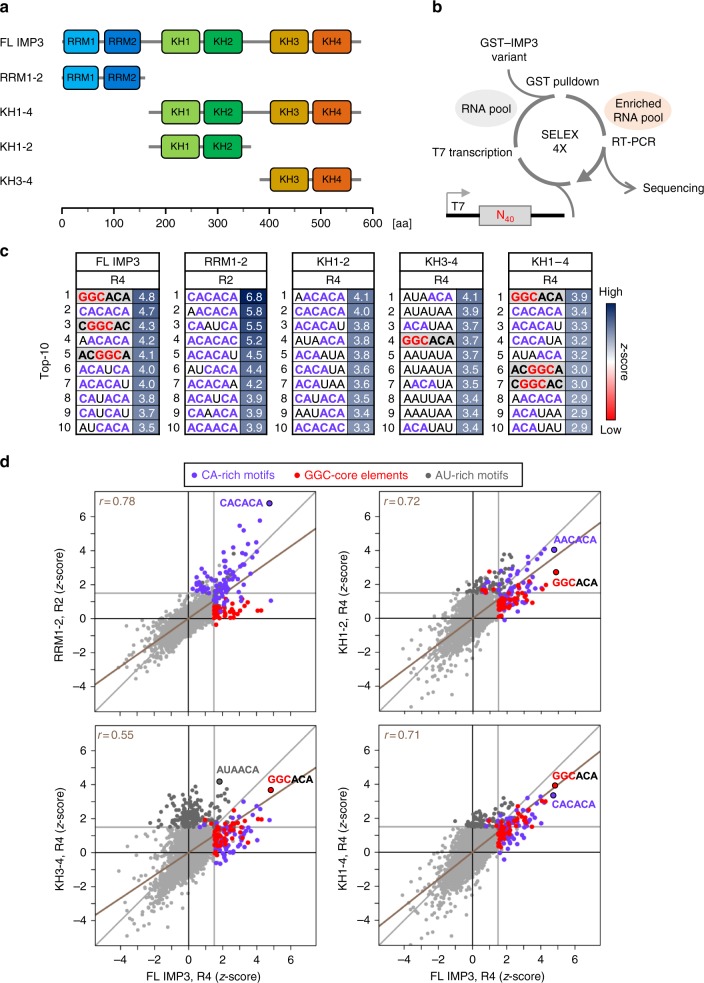
SELEX-seq analysis of IMP3 RNA-binding motifs. **a** Truncated IMP3 derivatives that were used for SELEX experiments (FL = full-length). RNA-binding domains are color-coded. **b** SELEX-seq procedure. Using GST-tagged IMP3 truncations (GST alone as negative and full-length IMP3 as positive control) and an N_40_-RNA pool, sequences bound by the respective proteins were enriched through four SELEX rounds and analyzed by sequencing after each round. **c** Top-10 enriched 6-mer motifs for all IMP3 derivatives measured by *z*-score after the fourth round of selection (R4), except for RRM1–2 (R2, for the complete dataset, see Supplementary Data 1). CA-rich motifs are highlighted in violet, elements with a common GGC consensus in red with gray background. **d** Correlation of 6-mer motif enrichment (measured by *z*-score) for IMP3 truncations (*y*-axis) in comparison with the positive control, full-length IMP3 (*x*-axis). Motifs with *z*-scores higher than 1.5 (vertical/horizontal gray lines) in either *x-* or *y*-axis are highlighted in violet for CA-rich motifs, red for GGC-core elements, and dark gray for AU-rich motifs. Pearson's correlation by linear regression is shown as a brown line with correlation coefficients (*r*) indicated

To dissect the complex RNA-binding properties of IMP3, we used individual, GST-tagged subdomains and applied an in vitro SELEX procedure, including four rounds of selection with a random N_40_-RNA pool and subsequent RNA-seq analysis (Fig. 1a, b, and Supplementary Fig. 1). Note that instead of standard short degenerate regions, we used an N_40_-RNA pool to be able to dissect and analyze arrays of several motifs, including their spacing; in addition, we sequenced after each round of selection, which allowed monitoring sequence enrichment throughout the SELEX procedure.

Single domains, such as RRM1 or KH1, did not show RNA-binding activity. In addition, previous structural studies had shown that at least the KH domains 3–4 of the related ZBP1/IMP1 are organized as a functional pseudo dimer (see the Introduction section). Therefore, we relied on truncated tandem domains for our analyses: RRM1–2, KH1–2, KH3–4, as well as an extended version containing all four KH domains, KH1–4 (Fig. 1a and Supplementary Fig. 1). In parallel, full-length IMP3 (as positive control) and GST alone (as negative control and for background correction) were analyzed. Motif-enrichment analysis by *z*-score calculation was performed for all possible 4-, 5-, and 6-mers, and were corrected at each round with the corresponding GST SELEX round (top-10 enriched 6-mer motifs in Fig. 1c; complete dataset in Supplementary Data 1). In parallel, the correlation of motif-enrichment datasets was tested for each tandem domain by comparison with the positive control, full-length IMP3 (Fig. 1d).

For the full-length IMP3 protein, this SELEX analysis revealed two populations of enriched motifs, CA-rich motifs as well as motifs with a GGC core (GGCA and CGGC; Fig. 1c). The KH1–4 variant, which lacks the N-terminal RRM domains, showed a very similar motif enrichment as the full-length protein, revealing that the four KH domains recognize both types of motifs (Fig. 1c, d). Separate analysis of KH1–2 and KH3–4 tandem domains also showed the enrichment of GGC-core elements within the top-30 hexamers (Supplementary Data 1), but the most-enriched sequences were either CA- (KH1–2) or CA/AU-rich (KH3–4), indicating that at least one of the KH domains of each tandem binds such a sequence (Fig. 1c, d, for the enrichment of AU sequences, in particular by KH3–4, see the Discussion section).

Most surprisingly, we found that RRM1–2, which until now had been described as nonfunctional in RNA binding, in fact exhibited a high preference for CA-rich and CA-repeat sequences, but not for the GGC-core elements (Fig. 1c, d). This specificity was observed after the second SELEX round, but was lost with more stringent washing conditions within rounds 3 and 4. Therefore, only the first two SELEX rounds were analyzed for the RRM1–2 derivative (see Discussion). Furthermore, a comparison of all SELEX rounds between the complete set revealed that, as expected, KH1–2, KH3–4, and the longer KH1–4 variant overlap the most, whereas RRM1–2 showed the least overlap with the isolated KH domains (Supplementary Figs. 1 and 2).

Taken together, our findings strongly argue for differential recognition of an extended array of two different types of motifs (CA-rich and GGC-core elements), which are bound by the KH tandem domains. Besides that, we provide evidence that the RRM1–2 domains contribute additional binding of a CA-rich element.

### A model for RNA recognition by IMP3

**Fig. 2:**
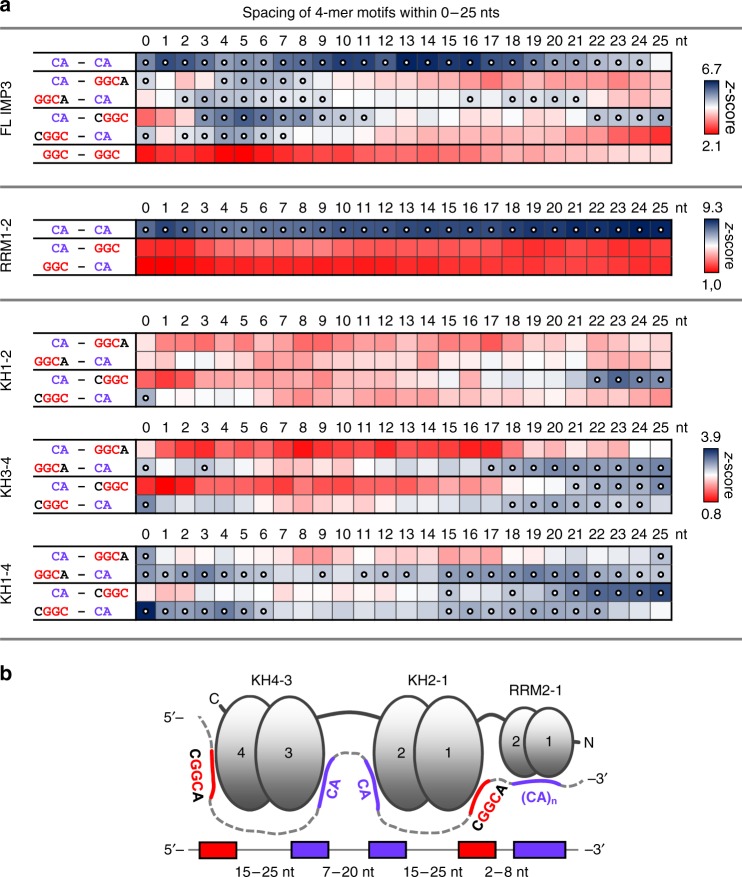
Spacing analysis reveals a consensus array of IMP3-binding motifs. **a** Enrichment of motif combinations with spacing between 0 and 25 nts for the full-length IMP3 (top), and RRM1–2 (middle), KH1–2, KH3–4, and KH1–4 domains (bottom), measured by a *z*-score and shown as a heat map. The combinations of the two GGC-core elements (GGCA/CGGC) with CA-rich motifs are shown for full-length IMP3 and the KH-containing derivatives, the combinations of two GGC-core elements (GGC/GGC) for full-length IMP3 only. Spacing between CA-rich motifs was analyzed for full-length IMP3 as well as RRM1–2 (for a summary of all combinations of CA-rich and GGC-core motifs, see Supplementary Data 2 and Methods). Individual *z*-score scales are given on the right. Positions with z-scores above the threshold used for description are indicated by circles (FL-IMP3 and RRM1–2: *z*-score >4.6; KH1–2, KH3–4, and KH1–4: *z*-score >2.5). **b** Model for RNA recognition by IMP3, based on SELEX-seq analysis

To identify how the different domains of IMP3 recognize consecutive elements on a single RNA, we analyzed our SELEX-seq data for spacing between enriched 4-mer motif combinations, using a window of 0–25 nts (Fig. 2a). Enriched combinations of two types of motifs (CA-rich and GGC-core elements) and their spacing were measured by *z*-score analysis (see Supplementary Data 2 and Methods).

Analysis of the full-length IMP3 data showed that the most-enriched motif combinations were either two CA-rich motifs with a short or medium-range spacing (CA-N_0–3_-CA; CA-N_7–20_-CA, with a maximum at N_13–16_), or a combination of a CA-rich motif with one of the identified GGC-core elements. For all combinations (CA-GGCA, GGCA-CA, CA-CGGC, and CGGC-CA), we observed shorter spacing of N_2–11_ nucleotides, with a maximum at N_4–6_. However, longer spacing was found to be clearly specific for either one of the two very similar GGC elements (GGCA versus CGGC): Only GGCA-N_18–21_-CA or CA-N_22–25_-CGGC were enriched, but not the respective reverse orientations (Fig. 2a, top). This indicates that, first, these sequence elements need to be appropriately spaced for recognition by IMP3; second, the arrangement of two motifs relative to each other is essential, and third, that both GGC-core elements seem to be differentially recognized. Finally, combinations of two GGC elements were, in comparison, not enriched.

Next, we applied this approach to the KH subdomains to obtain a refined view of motif spacing for IMP3. For each of the KH1–2, KH3–4, and KH1–4 subdomains, we analyzed spacing between either one of the two GGC-core elements (GGCA versus CGGC), and the respective combination with CA-rich motifs identified through analysis of the full-length protein (Fig. 2a, bottom).

Strikingly, we found that the KH1–2 subdomain shows a preference only for the combination of CA-rich motifs and the CGGC element in one of the possible orientations, with a CA-N_22–25_-CGGC spacing optimum. At the same time, we observed no selection of the three other combinations, underlining a high specificity for both the relative arrangement of CA and GGC motifs, as well as for one type of GGC-core element (CGGC). This observation is supported by the results obtained for the full-length IMP3 protein (Fig. 2a, top).

In contrast, KH3–4 showed the strongest enrichment for GGCA-N_17–25_-CA, but—to a similar extent—appears to recognize also CGGC in combination with a CA-rich motif, in either orientation and with a spacing of N_21–25_ and N_18–24_, respectively. Similar to full-length IMP3 and KH1–2, the CA-GGCA motif combination was found to be least enriched for KH3–4.

Finally, for KH1–4, we detected a mix of enriched motif spacing already observed for the separate KH1–2 and KH3–4 domains, with a preference for both GGCA-N_15–25_-CA and CA-N_20–25_-CGGC orientations, but also for CGGC-N_15–22_-CA (Fig. 2a, bottom; see Discussion). For all tested KH subdomains, enrichment of shorter spacing was observed specifically in the case of GGCA-CA and CGGC-CA combinations (KH1–2: N_0_, KH3–4: N_0–3_, and KH1–4: N_0–6_), most likely representing a 3′-CA extension of these motifs rather than real spacing, since previously published data argue for a minimal spacing requirement of N_10–25_ between two motifs recognized by a KH di-domain.

In addition, spacing analysis for RRM1–2 revealed strong enrichment for CA-rich motif combinations in all positions within the 25- nts window, but not for the GGC-core elements (Fig. 2a, middle), again arguing for a high preference for extended CA-rich repeat elements, in agreement with our previous analyses (Fig. 1c, d, see Discussion). As mentioned above, we also observed shorter spacing between N_2–11_ for GGC and CA elements in both orientations within the full-length context of all six RBDs (FL-IMP3). While a mixture of spacing/orientations for all domains is expected, a comparison with KH1–4 argues that specifically shorter spacing reflects the influence of RRM1–2. Therefore, we interpret this as spacing between a GGC motif bound by one of the KH domains and a nearby CA element recognized by RRM1–2.

Based on these datasets, we assembled a working model of how IMP3 recognizes RNA (Fig. 2b). Due to the selective enrichment of specific motif arrangements and the known sequence preference of KH3–4 subdomains of the IMP1 paralog (see Introduction), we propose that KH1 and KH4 each recognize sequence elements with a common GGC core, whereas KH2 and KH3 bind to CA-rich motifs. The RRMs may provide an additional, stabilizing interaction with adjacent CA-rich motifs. It should be noted that due to the symmetry of this array of sequence elements, our spacing analysis would partially support both polarities of IMP3 binding to its target RNAs.

### In vitro analysis of IMP3 RNA recognition

**Fig. 3:**
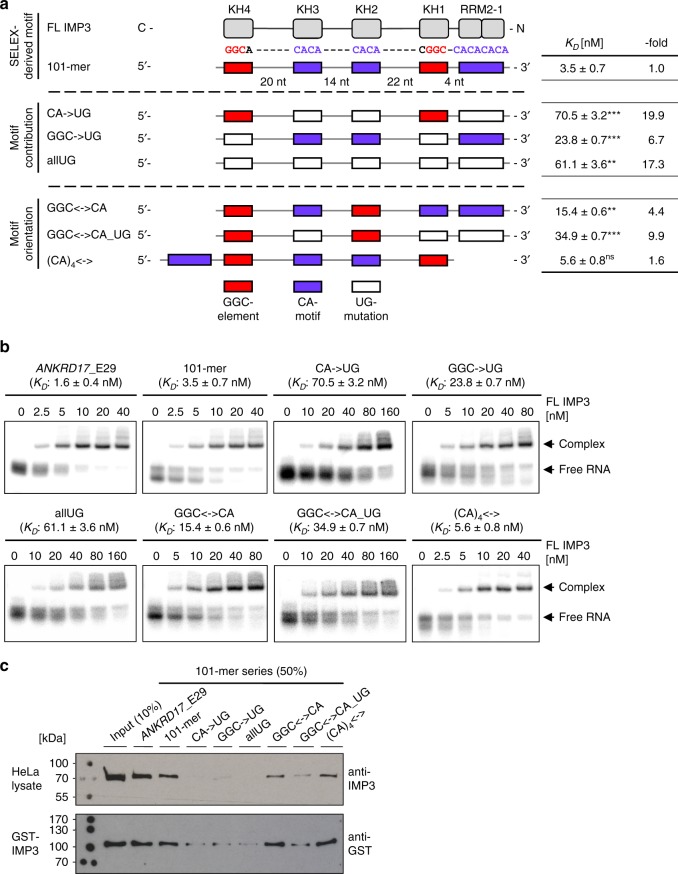
Validation of the SELEX-derived array of IMP3-binding motifs: mutational analysis. **a** Design of a 101-mer RNA, containing all SELEX-derived IMP3-binding motifs (GGC motifs, red boxes; CA motifs, violet boxes) with appropriate spacing and serving as a basis for mutational analysis and validation assays. The IMP3 domains potentially interacting with the respective sequence elements of the 101-mer RNA are indicated (top). The contributions of specific motifs were tested by mutational analysis (CA motifs or GGC-core elements or both of them mutated to UG, middle). The importance of motif orientation was analyzed by shuffling of domain-specific sequence motifs (KH1–2-specific motifs: GGC<->CA; additional substitution of the CA motifs: GGC<->CA_UG; relative positioning of the RRM1–2-specific motif: (CA)_4_<->, bottom). *K*_D_ values obtained by electromobility shift assays (EMSAs, see panel **b**) and the respective changes in binding affinity (-fold) compared with the wild-type 101-mer sequence are summarized on the right (*p* < 0.005^**^, *p* < 0.001^***^, ns = not significant, two-sided *t* test). **b** IMP3 interaction with RNAs of the 101-mer series, assayed by EMSAs. Full-length protein (0–40, 0–80, or 0–160 nM) was titrated to a constant concentration of the respective ^32^P-labeled RNAs (5 nM, mean and standard error of three experiments). A 121-nt region from the IMP3 target mRNA *ANKRD17* (exon 29) served as a positive control. For the corresponding binding curves, see Supplementary Fig. 3. **c** Pulldown of endogenous IMP3 in HeLa cell lysate (top) or of recombinant GST–IMP3 (bottom) with 3′-biotinylated RNAs of the 101-mer series. IMP3 was detected by western blot with either IMP3- (top) or GST-specific antibodies (bottom). Source data are provided as a Source Data file

To test our working model presented in Fig. 2b, we designed an RNA sequence based on our SELEX analysis, containing domain-specific minimal 4-mer sequence elements that are appropriately spaced by unrelated sequences, extending to a total length of 101 nts (101-mer RNA): GGCA-N_20_-CACA-N_14_-CACA-N_22_-CGGC-N_4_-(CA)_4_ (Fig. 3a, for the full sequence, see below and Supplementary Data 3).

The 101-mer RNA was used as a basis for mutational analysis to determine the contribution of individual sequence elements to the overall affinity of the protein. Electromobility shift assays (EMSAs) revealed that the full-length protein recognizes the ^32^P-labeled 101-mer RNA with high affinity (dissociation constant *K*_D_ = 3.5 ± 0.7 nM, Fig. 3a, b and Supplementary Fig. 3), comparable to the positive control, a sequence of similar length derived from exon 29 of the *ANKRD17* transcript (121 nts, *K*_D_ = 1.6 ± 0.4 nM, Fig. 3a, b). The *ANKRD17* transcript had been recently identified by us as strongly IMP3-associated^27^ and harboring nearly the exact array of sequence elements proposed in our 101-mer. Note that RNA secondary structure predictions using the Vienna RNAfold server^31^ revealed that in the wild-type and mutant 101-mers, the proposed short motifs are mainly present as linear elements or involved in base pairing with less than 50% probability. On average, we find the minimum free energy structures to be represented with maximally 22% of all structures of a possible thermodynamic ensemble, while ensembles are very diverse. Altogether, this poses a high degree of accessibility for IMP3 to the RNA-target elements. In line with that, previous studies report a significantly lower degree of RNA secondary structure in vivo compared with in vitro, including active RNA unfolding^32^.

To test for motif contribution within the 101-mer sequence, we either substituted the CA motifs (CA->UG), the GGC-core elements (GGC->UG), or a combination of both (allUG), each by mutating to UG (for full sequences, see Supplementary Data 3). Substitution of the GGC-core elements led to a seven-fold reduction in affinity, and mutation of the CA motifs, or a combination of both, led to a 17- to 20-fold reduction (Fig. 3a, b). This indicates that both elements are important for high-affinity RNA recognition.

We also evaluated the importance of motif orientation, by changing the order of the presumably KH1–2-specific elements (GGC<->CA), resulting in a four-fold decrease in affinity (Fig. 3a, b). The additional substitution of CA motifs within this context (GGC<->CA_UG) led to a further reduction (10-fold). This shows that the protein prefers the SELEX-derived orientation of elements, but can adapt to changes with relatively modest effects on binding affinity. Furthermore, we tested the influence of the CA-repeat element, which is located on the very 3′ end and—based on our model—expected to be contacted by RRM1–2, by moving it to the 5′ end ((CA)_4_<->). Surprisingly, the binding affinity remained unchanged, suggesting that either this element does not significantly contribute to the overall affinity or that IMP3 can recognize the element in both positions, consistent with our spacing analysis (see Fig. 2).

To address the stoichiometry of the major RNA–protein complex observed here and in the following assays, we also compared complex formation with full-length IMP3 proteins with or without GST tag, as well as with an equimolar mixture of both of them (Supplementary Fig. 4). Since in the latter case we did not observe a complex of slower mobility, there appears to be no complex with two copies of IMP3 per RNA, supporting a 1:1 stoichiometry of IMP3 complex formation. Finally, GST by itself did not bind RNA, and the GST tag affected IMP3 complex formation only to a minor level, and that only at the highest concentrations.

Our EMSA-based results were consistent with pull down assays of endogenous IMP3 protein from HeLa cell lysate as well as of recombinant GST-tagged IMP3 with 3′-biotinylated RNAs and subsequent Western blot detection (Fig. 3c).

In sum, these consistent results from biochemical assays, quantitative EMSA, and semiquantitative pulldown strongly support our proposed model of target RNA recognition involving all IMP3 RBDs (Fig. 2b).

### Structure and RNA recognition by the IMP3 tandem KH1–2 domain

**Fig. 4:**
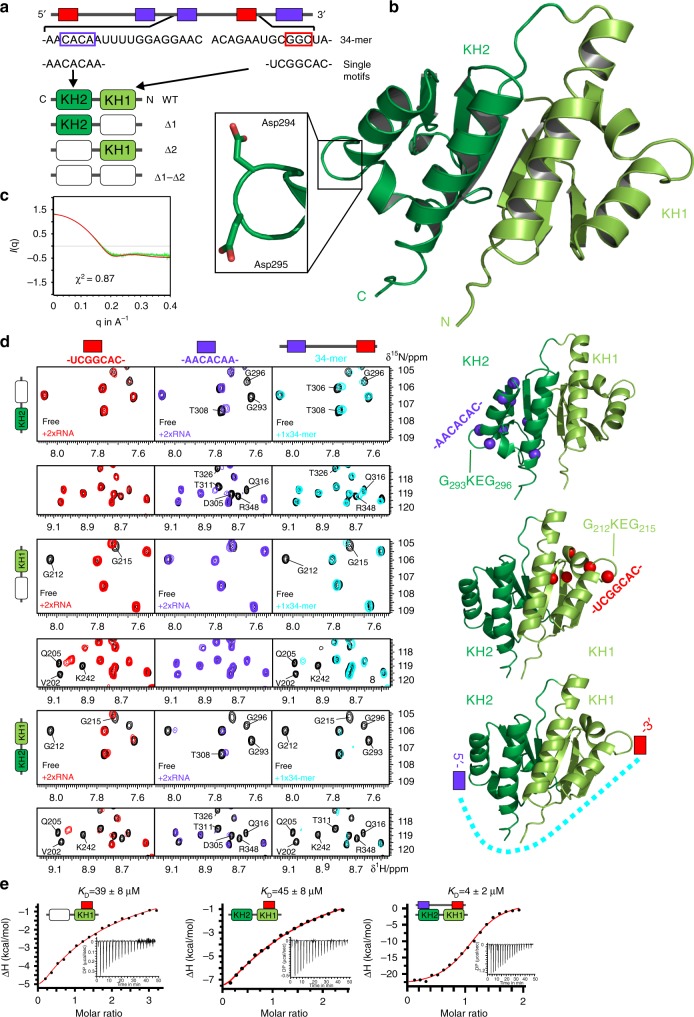
Structure and RNA recognition of the IMP3 tandem KH1–2 domain. **a** Protein constructs and RNAs used. (Top) Scheme of the 101-mer RNA region, which includes the 34-mer sequence (below), covering the cognate binding region of the KH1–2 domain. The two recognition sequences for KH1 and KH2 are embedded in two respective 7-mers. (Bottom) Wild-type (WT) and three different versions of KH1–2 (in Δ versions of the domains, GKEG was replaced by GDDG^30^). A proof of concept for this approach is shown in Supplementary Fig. 5. **b** Crystal structure of the KH1–Δ2 tandem domain (see also Supplementary Table 1 and Supplementary Fig. 5). The zoom-in shows the mutated GKEG loop with two aspartates replacing Lys294 and Glu295 in KH2. **c** SAXS curve of KH1–Δ2 at 4 mg/ml and overlaid with a theoretical curve from the crystal structure in **b** created by *Crysol* (red)^63^. **d** HSQC overlays showing KH1–2 versions Δ1 (upper), Δ2 (middle), and WT (lower row) free (black) and when bound to twofold excess of either of the short RNAs or equimolar 34-mer RNA (see color code). Two different spectral regions (top/bottom) are shown. Selected residues as representative probes in the active subdomains (light/dark green color for KH1 and KH2, respectively), are annotated in the spectra. Amide groups of strongly affected residues are shown as spheres in the structures on the right. The scheme at the lower right suggests the mode of KH1–2 interacting with the 34-mer RNA. Complete NMR spectra and CSP plots are provided in Supplementary Fig. 6 and 7. **e** Representative ITC curves for binding of KH1 (in the KH1–Δ2 context) and KH1–2 WT when titrated with UCGGCAC. The plot on the right shows the binding of KH1–2 WT to the 34-mer RNA comprising both motifs. The suggested topology of the protein–RNA complex and dissociation constants (*K*_D_) for the interaction are indicated (mean and standard deviation of three experiments). All ITC measurements are summarized in Supplementary Table 2. Source data are provided as a Source Data file

Given substantial primary sequence conservation of the IMP1 and IMP3 KH3–4 tandem domains (Supplementary Fig. 4), similar RNA-binding features were expected for IMP3 KH3–4, as suggested by Chao and colleagues^14^. In contrast, the RNA recognition by the IMP3 KH1–2 tandem had so far not been analyzed. To determine the individual contributions of KH1 and 2 (Lys192 to Ile355), their RNA binding was inactivated by mutation (GKEG motif to GDDG), while maintaining the crucial tandem context^14–16^, resulting in four possible combinations (Fig. 4a). Our NMR data clearly proved the integrity of all constructs (Supplementary Fig. 5). We analyzed crystals of both wild-type KH1–2 and KH1–Δ2 versions for structural characterization. While the former only generated very low-resolution diffraction data, we were able to solve the structure of KH1–Δ2 at 2.15-Å resolution (Fig. 4b and Supplementary Table 1). SAXS (small-angle X-ray scattering) data back-calculated based on the crystal structure are in good agreement, indicating that the crystal structure reflects the monomeric solution geometry (Fig. 4c), which also closely resembles other tandem KH domains (Supplementary Fig. 5). We conclude that the IMP3 KH1–2 tandem is a stable monomeric folding unit.

We next examined RNA-binding contributions of the KH1 and KH2 domains by inactivation of the individual domains in the KH1–2 context, using SELEX-derived 7-mers from the rationally designed 101-mer (Figs. 3, 4a, d and Supplementary Figs. 6 and 7). First, NMR was used to identify the RNA sequence recognized by the individual subdomains (Fig. 4d). Indeed, KH1 clearly favors binding of the GGC motif, while KH2 prefers binding to the CA-RNA. We did not see any considerable cross-reactivity of domains with the respective unrelated RNA in the context of single KH1–2 Δ versions, as shown by a full CSP analysis (Supplementary Figs. 6 and 7).

Can we also observe specific binding of motifs in the wild-type KH1–2 context? Here, a clear preference of KH1 for its GGC target motif was observed, while KH2 showed a lower, but significant preference for CA. Given that larger NMR CSPs were observed for the KH1/GGC, compared with the KH2/CA-RNA interaction, RNA binding appears to be mediated primarily through KH1. Indeed, ITC revealed a measurable KH1–GGC interaction in the low-to-medium micromolar range, while the KH2–CA complex could not be determined in our ITC setup (Fig. 4e and Supplementary Table 2). Notably, the respective interactions were also observed in the context of the intact wild-type KH1–2.

When both the GGC and the CA-RNA motifs are present in a single RNA ligand, an overall higher binding affinity for wild-type KH1–2 is expected. To confirm this, we used a corresponding region (34-mer, Fig. 4a) from the 101-mer RNA, including a 22-nt linker separating the GGC- and CA motifs, as suggested by the spacing analysis (Figs. 2, 3a). As shown in Fig. 4d, significant CSPs were observed for KH1 and KH2 that compare well with the titration with short 7-mer GGC- and CA-RNA sequences, respectively. However, spectral changes in general appeared to be more widespread. In HSQC experiments, we observed severe line broadening for most NMR signals in either subdomain upon titrating the 34-mer RNA (Fig. 4d and Supplementary Figs. 5, 6, and 7). This is in line with an increase in molecular weight caused by the RNA and affecting major parts of KH1–2, suggesting a compaction of the complex. The simultaneous recognition of both RNA motifs in a 1:1 complex requires looping of the 34-mer RNA around the KH1–2 tandem (Fig. 4d). Despite a lack of clear evidence of the N–C versus 5′−3′ polarity of individual KHs to their RNA motifs in our study, previous studies reason the orientation of the RNA loop to run in parallel with residues connecting the two KH domains^14–16^. Referring to that, the suggested scheme in Fig. 4d is in line with our proposed model of the relative IMP3–RNA alignment (Fig. 2b).

Finally, we performed ITC experiments with the wild-type KH1–2 and 34-mer RNA (Fig. 4e and Supplementary Table 2). As expected, a 10-fold higher affinity compared with the single interactions of 7-mer RNAs indicates a cooperative binding event that shifts affinity by one order of magnitude. The 1:1 stoichiometry of the KH1–2/34-mer RNA complex clearly argues for the formation of a looped-RNA–KH1–2 complex, which is also supported by a significant gain in the entropy term. Altogether, our data support the preference of KH1–2 subdomains for specific SELEX-derived RNA motifs and cooperative recognition when both motifs are present in a longer context.

### Molecular determinants of IMP3 RRM1–2–RNA interactions

**Fig. 5:**
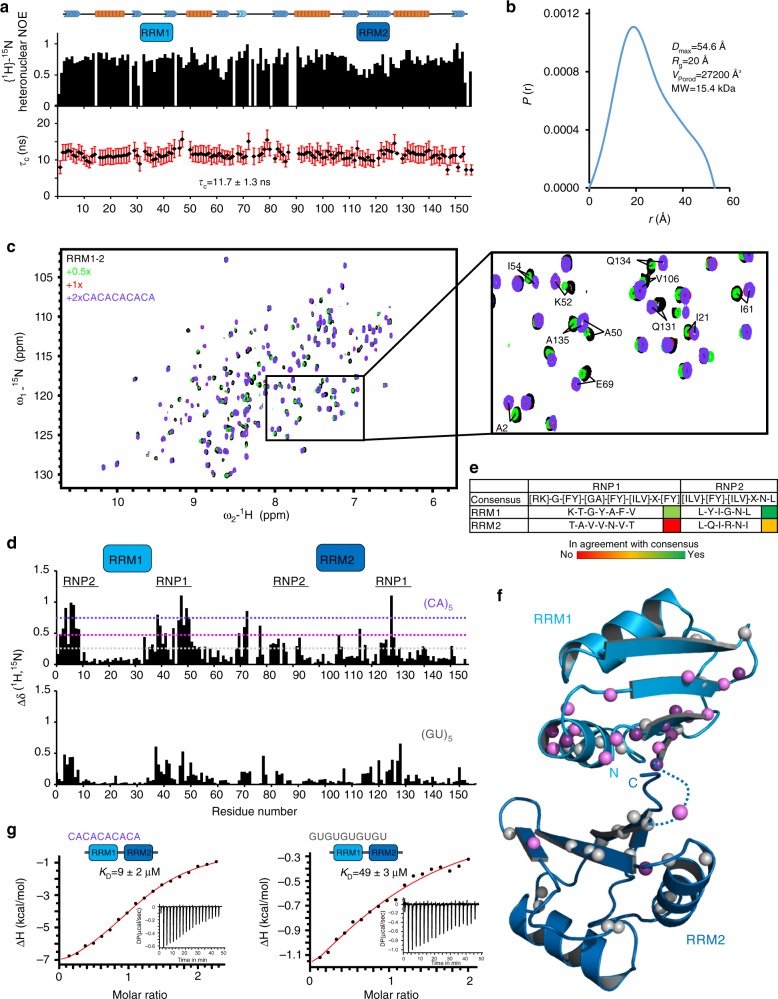
RNA recognition mode of the IMP3 RRM1–2 tandem domain. **a** IMP3 RRM1–2 function as tandem in solution. Secondary structure elements in the RRM1–2 tandem domain as obtained from secondary chemical shifts are shown on top. {^1^H}–^15^N heteronuclear NOE values show that the linker connecting the two globular domains is rigid. Tumbling correlation-time values (τ_C_, bottom), derived from NMR relaxation data (Supplementary Fig. 8), show an average value of 11.7 ns, indicating that both domains tumble together in solution. Gaps indicate prolines or residues with missing data. Error bars derived from error propagation using T_1_ and T_2_ values in Supplementary Fig. 8. **b** Pairwise distance distribution, P(*r*), for IMP3 RRM1–2 at 1 mg/ml derived from SAXS data (Supplementary Fig. 8). The maximum pairwise distance (*D*_max_), radius of gyration (*R*_g_), and the Porod volume (*V*_Porod_) are consistent with a monomeric RRM1–2 tandem domain particle in solution. **c** Overlay of ^1^H,^15^N NMR correlation spectra of RRM1–2 alone and in the presence of different concentrations of (CA)_5_ RNA (see color code). The inset shows representative residues affected by RNA binding. **d** Chemical shift perturbations (CSP) observed (see panel **c**) at the endpoint of the titration. The two domains and their RNP sequence motifs are labeled on top. The dotted lines indicate CSP thresholds calculated as average (gray) plus one and two standard deviations (pink and violet, respectively). The lower panel shows CSP from an NMR titration with (GU)_5_ RNA (Supplementary Fig. 8). **e** RNP sequence motifs in the RRM1 and RRM2 subdomains. **f** Mapping of CSPs for the titration with the (CA)_5_ RNA (**d**) onto a structural model of RRM1–2 (see the Results and Methods sections). Amides are shown as spheres colored according to thresholds in (**d**). **g** ITC data for the titration of RRM1–2 with (CA)_5_ or (GU)_5_ RNAs. A titration of (CA)_3_ hexamer to RRM1–2 is shown in Supplementary Fig. 8. The suggested complex topology and *K*_D_ values are indicated. Values represent mean and standard deviation of three experiments. All ITC measurements are summarized in Supplementary Table 2. Source data are provided as a Source Data file

To assess the RNA interactions of the IMP3 RRM1–2 domains, we purified an optimized construct, which yields excellent NMR spectra, consistent with a monomeric conformation. Secondary chemical shifts reveal the presence of a canonical RRM secondary structure (Supplementary Fig. 6). NMR ^15^N relaxation experiments indicate a compact arrangement of domains with almost no linker flexibility, suggesting that the two domains appear as tandem (Fig. 5a). This is also supported by the tumbling correlation time, estimated from ^15^N *R*_1_ and *R*_2_ relaxation rates, consistent with a globular 18-kDa protein (Fig. 5a and Supplementary Fig. 8). Static-light scattering unequivocally proves the protein to be a monomer (Supplementary Fig. 8). SAXS data indicate a compacted arrangement of the tandem domains (Fig. 5b).

We next tested binding of CA-repeat RNAs by RRM1–2 using NMR titrations. A (CA)_5_ 10-mer was chosen to potentially cover both RRMs (Fig. 5c). The majority of significant CSPs localizes to RRM1, while only a few amides in RRM2 still showed CSPs above average. Hot spots map to regions around the RNP motifs (Fig. 5d). Interestingly, the control RNA, (GU)_5_, led to a very similar, yet much weaker pattern of CSPs in RRM1 and 2, indicating a preference for CA.

Sequence analysis suggested that RRM2 harbors a degenerate RNP2 motif and lacks a canonical RNP1 motif (Fig. 5e). We conclude that CSPs in RRM2 were observed because they are indirectly affected by RNA binding in RRM1 and caused by the length of the RNA. We repeated NMR titration experiments of RRM1–2 with a (CA)_3_ 6-mer RNA that should not extend toward RRM2 in the tandem domain arrangement. However, we found almost identical CSPs (Supplementary Fig. 8) as compared with (CA)_5_, which suggests that the two domains are arranged in a way that causes binding of RNAs through RRM1 to be sensed by nearby residues in RRM2. We derived a structural model of the RRM1–2 tandem domains filtered against SAXS data and NMR CSPs (see Methods) (Fig. 5f and Supplementary Fig. 8). The model shows a compacted arrangement of RRM1 with RRM2 in a unique spatial orientation of tandem RRMs that requires the RRM1-bound RNA to pass the RRM2 β-sheet and potentially also involve linker residues. Chao and colleagues very recently succeeded in obtaining the crystal structure of RRM1–2^33^. Notably, this structure agrees very well with our model with an RMSD value of 4.4 Å for the overall RRM1–2 arrangement, suggesting that the linker indeed acts in stabilizing the RRM1–RRM2 interface. It also supports our data that only RRM1 accounts for RNA recognition and prefers CA-rich sequences.

Finally, ITC was used to quantify RNA binding to RRM1–2 (Fig. 5g and Supplementary Table 2). The interaction with (CA)_5_ revealed a low-micromolar affinity, and in line with our NMR data, we found the same affinity for RRM1–2 when binding to the 6-mer CA-RNA (Supplementary Fig. 8). This supports our hypothesis where binding takes place primarily in RRM1 through an interface with not more than six nucleotides of RNA. A five- to six-fold lower affinity of (GU)_5_ with RRM1–2 is consistent with the reduced CSPs. However, this number still shows some nonspecific RNA binding to this non-cognate motif, as often observed for canonical RRM- and KH domains^34,35^.

In sum, we have shown that RRM1–2 significantly contributes to the overall RNA binding of IMP3 through the specific recognition of CA-rich RNAs, as suggested by our SELEX experiments.

### All tandem domains of IMP3 contribute to RNA recognition

**Fig. 6:**
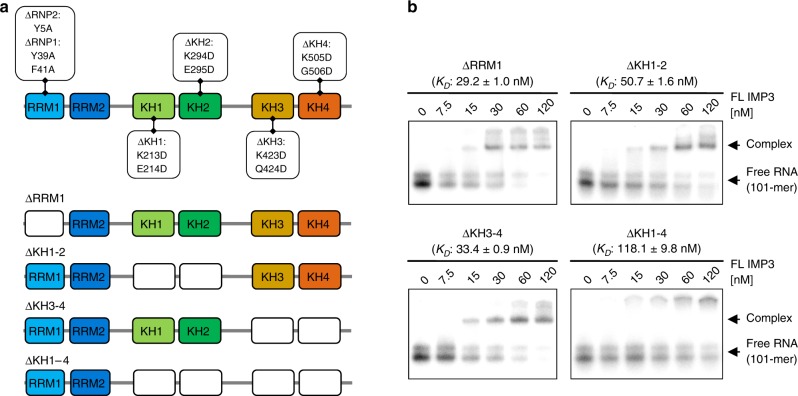
Functional analysis of individual RNA-binding domains of IMP3. **a** Summary of mutations introduced in full-length IMP3 for functional analysis of individual RNA-binding domains (top) and schematic representation of the resulting mutants used for binding assays (bottom). RRM1 was inactivated by mutation of critical aromatic RNP residues, whereas the KH domains were inactivated by GxxG to GDDG conversion^30^. **b** EMSAs of the IMP3 mutants with the SELEX-derived 101-mer RNA (see Fig. 3a). Mutated IMP3 derivatives (0–120 nM) were titrated to a constant concentration of ^32^P-labeled 101-mer RNA (5 nM, mean and standard error of three experiments). For the corresponding binding curves, see Supplementary Fig. 3. Source data are provided as a Source Data file

To further verify the suggested concept with all IMP3 RBDs engaged in multivalent RNA recognition, we next tested the contribution of individual tandem domains within the full-length-protein context. Therefore, we mutated critical amino acids in the respective domains to inactivate individual tandem domains (ΔRRM1, ΔKH1–2, ΔKH3–4, and ΔKH1–4; Fig. 6a), followed by EMSA assays with the designed 101-mer RNA (Fig. 6b). Since RRM2 does not contain well-conserved RNP motifs and consistent with our structural analysis (Fig. 5), only RRM1 of the RRM1–2 tandem domain was mutated to assess the contribution of the RRM1–2 tandem domains^33^. Strikingly, inactivation of RRM1 alone led to an eight-fold reduced affinity compared with wild type (WT), indicating that this domain indeed contributes to RNA binding also in the full-length context.

Inactivation of the KH3–4 tandem domains also reduced affinity to approximately nine-fold, and ΔKH1–2 showed the strongest effect with a 14-fold decreased affinity. These still rather mild effects probably reflect the complex contribution of all tandem domains to the overall affinity. Specifically at low protein concentrations, fitting three of the four tested mutants required Hill coefficients >1, indicating apparent cooperativity. We believe that these effects likely reflect different fractions of active protein, loss of protein, or protein aggregation due to introduced mutations (Supplementary Fig. 3c). Only mutation of all four KH domains (ΔKH1–4) led to a near-complete loss of binding activity. However, note that the observed ΔKH1–4 complexes did not enter the gel, arguing for aggregation of ΔKH1–4 (Fig. 6b).

Taken together, this mutational analysis provides further evidence that all tandem RNA-binding domains of IMP3 actively contribute to RNA recognition.

### SELEX-derived IMP3 consensus in endogenous RNAs

Our findings suggest that IMP3 binds to a complex array of multiple sequence elements, composed of CA- and GGC elements with certain spacing constraints that can extend over more than 100 nts. To test whether our SELEX-derived motif array describes in vivo IMP3 RNA binding, we determined whether iCLIP tags are more densely located in 3′-UTRs containing the motif array than in those with no motif array (for parameters of motif array search and iCLIP assays in HepG2 cells, see Methods). Such a correlation approach may also be valuable to predict IMP3 targets.

**Fig. 7:**
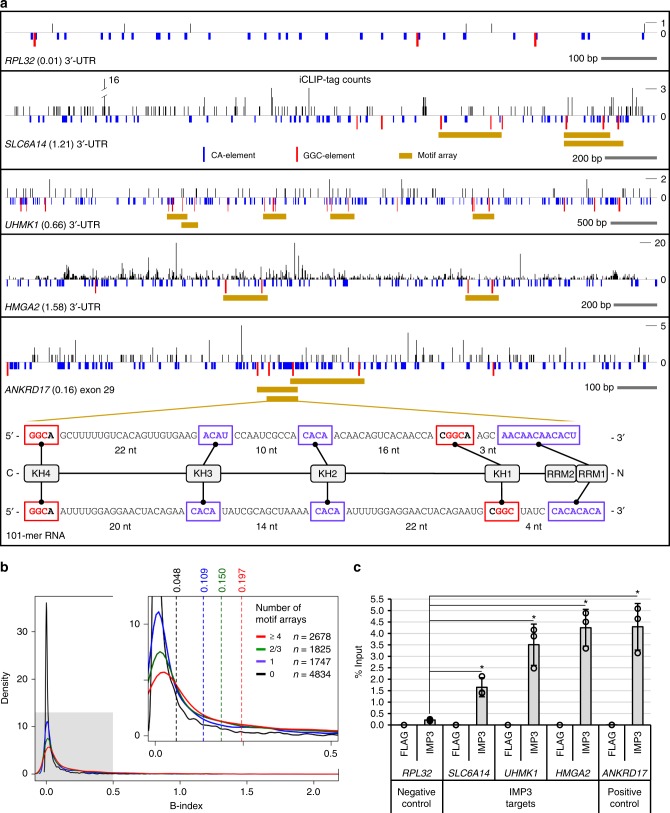
SELEX-derived consensus array in natural IMP3 targets. **a** For four selected 3′-UTRs (*RPL32* as a negative control and *SLC6A14*, *UHMK1*, and *HMGA2* as IMP3 targets) and exon 29 of *ANKRD17*, the iCLIP-tag distribution of IMP3 in HepG2 cells (above the horizontal lines) and the distribution of CA-/GGC elements (in violet/red, below the lines) are schematically represented. In addition, the positions of motif arrays (in brown) are indicated. Binding indices are given in parentheses, reflecting normalized iCLIP-tag densities in the respective 3′-UTRs. For one of the motif arrays of *ANKRD17* exon 29, a detailed sequence comparison with the optimal, designed motif array in the 101-mer RNA is shown. **b** Global correlation of IMP3 RNA binding and motif array distribution, represented as a density plot of B index for 3′-UTRs grouped by the number of motif arrays (0, 1, 2/3, and ≥4). In the enlarged segment, the mean values of B indices and the number of 3′-UTRs in each data group are given. **c** Validation of IMP3 binding, using RIP assays from HepG2 lysates (anti-IMP3 and anti-FLAG antibodies), followed by RT-qPCR assays for predicted IMP3 targets (*SLC6A14*, *UHMK1*, and *HMGA2*), with *RPL32* as negative, and *ANKRD17* as positive control (statistical deviations based on biological triplicates; *p* < 0.05^*^, two-sided *t* test). Source data are provided as a Source Data file

Using HepG2 whole-cell polyA+RNA-seq data (ENCODE/CSHL) as an expression reference, iCLIP-tag counts in each 3′-UTR, normalized by the respective expression levels, were summarized to yield an index of in vivo binding (B index). Figure 7a shows how iCLIP-tag counts (represented above the horizontal lines for each target) and CA-/GGC elements, as well as complete arrays (below the lines) distribute over four selected 3′-UTRs: *RPL32*, as a negative control, with a very low B index (0.01) and containing no motif array; *SLC6A14* and *UHMK1* as two examples of predicted IMP3 targets (B indices: 1.21 and 0.66, respectively); and *HMGA2*, a known IMP3 target (B index: 1.58). In addition, we had previously identified and validated *ANKRD17* exon 29 as an IMP3 target that is not only spliced in the canonical mRNA, but also additionally processed into a circular RNA^27^. Analysis of the sequence and iCLIP-tag counts also predicted *ANKRD17* exon 29 as an IMP3 target (B index: 0.16), with one of the motif arrays exhibiting a motif-spacing pattern very similar to our rationally designed 101-mer RNA (see bottom panel).

On a global level, from the total set of 11,084 3′-UTRs expressed in HepG2 cells, 4834 (44%) contain no motif array, 1747 (16%) contain one, 1825 (16%) two or three, and 2678 (24%) at least four arrays (Fig. 7b). The distribution of B indices for motif-array-containing 3′-UTRs is higher than that for 3′-UTRs without motif arrays, as the density plots show. The higher the number of motif arrays, the higher are the mean values of B indices and their significance (p-value of Welch two-sample t test: 1.56–e29, 3.52e–53, and 1.11e–95, comparing 3′-UTRs with 1, 2/3, and 4 motif arrays, respectively, with 3′-UTRs with no motif array). This confirms a clear correlation between IMP3 in vivo binding and our SELEX-based IMP3 RNA-binding motifs.

Finally, both predicted IMP3 targets (*SLC6A14* and *UHMK1*) were positively validated (Fig. 7c), using RNA-immunoprecipitation (RIP) assays from HepG2 lysates with anti-IMP3 antibodies (anti-FLAG as a control), followed by RT-qPCR assays for the respective mRNAs (*RPL32* as negative, and *HMGA2* and *ANKRD17* as positive controls). This was further validated by quantitative EMSA with an isolated region from the 3′-UTR of the well-studied *IGF2* mRNA (see Supplementary Fig. 9).

In sum, our results strongly support the biological significance and the predictive value of our SELEX-derived model for IMP3-RNA recognition of extended motif arrays that can reside in either 3′-UTRs or coding sequences.

### IMP3 interferes with let-7-mediated repression of *HMGA2* mRNA

**Fig. 8:**
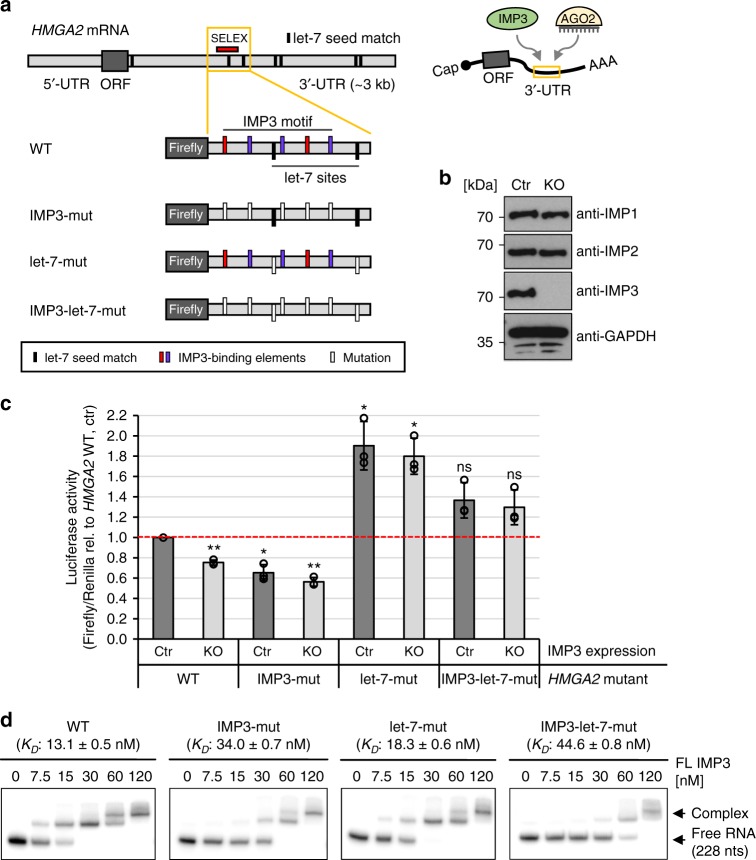
Cross-regulation of *HMGA2* mRNA expression by let-7 miRNA and IMP3. **a** Schematic of the *HMGA2* mRNA, indicating the seven let-7 miRNA seed matches (black bars) in the 3′-UTR and the SELEX-consensus array (red bar). Below, the structures of luciferase wild-type (WT) and mutant reporters are given, containing the *HMGA2* 3′-UTR region (yellow box) with the IMP3 SELEX-consensus array and two let-7 seed matches. To measure the effect of IMP3 binding, IMP3-binding elements were mutated (IMP3-mut, GGC/CA ->UG, red/violet bars); for analysis of the let-7 influence, the two seed matches in this region were inactivated (let-7-mut, UACCUCA ->UAaCgCA, black bars). In addition, both mutations were combined (IMP3-let-7-mut). On the right, binding of IMP3 and the let-7-AGO complex within the *HMGA2* 3′-UTR is schematically represented. **b** Western blot analysis of standard (ctr) and CRISPR/Cas9 genome-engineered IMP3-knockout (KO) ES-2 cells, detecting endogenous levels of IMP1, IMP2, and IMP3. GAPDH was used as loading control. **c** Standard (ctr) and IMP3-knockout (KO) ES-2 cells were transfected with luciferase constructs described in **a**. Luciferase activities were measured as a ratio of Firefly/Renilla activity and compared with control cells transfected with the *HMGA2* WT construct (statistical deviations based on biological triplicates; *p* < 0.005^**^, *p* < 0.001^***^, ns = not significant, two-sided *t* test). **d** EMSA assays with ^32^P-labeled *HMGA2* mutant RNAs (0–120 nM IMP3 and 5 nM RNA; mean and standard error of three experiments, Hill coefficients of *n* = 3.1 ± 0.3 for WT, *n* = 6.0 ± 0.8 for IMP3-mut, *n* = 4.7 ± 0.6 for let-7-mut, and *n* = 3.5 ± 0.2 for IMP3-let-7-mut), containing the SELEX motif and a single let-7 seed sequence (see red bar in **a**). Source data are provided as a Source Data file

Analysis of our iCLIP data had revealed that *HMGA2*, a well-known IMP-regulated mRNA, harbors the IMP3-binding site within a region that also contains two let-7 miRNA seed sequences (Fig. 8a, yellow box). As previously reported^9^, a similar, overlapping region is targeted by IMP3, thereby interfering with let-7-dependent *HMGA2* mRNA destabilization. To functionally corroborate our analysis of IMP3 RNA-binding characteristics, we inserted this *HMGA2* region (266 nts) into a luciferase reporter construct and measured the effect of IMP3 motif mutations, let-7 seed mutations^11^, and a combination of both on relative luciferase activity (Fig. 8a). The respective luciferase reporter constructs were transfected either in standard ES-2 cells (ctr) or in CRISPR/Cas9 genome-engineered IMP3-knockout cells (KO) (Fig. 8b).

In comparison with the WT *HMGA2* sequence, where ~25% reduction in luciferase activity was observed in IMP3-KO cells, mutation of the IMP3 motif had a more pronounced effect (35% reduction in IMP3-expressing and 45% reduction in IMP3-KO cells), indicating functional inactivation of the IMP3-binding site (Fig. 8c). In contrast, mutation of the two let-7 seed sequences increased luciferase activity in both standard and IMP3-KO cells, reflecting the let-7-dependent negative regulatory effect. In addition, by combining both mutations (IMP3-let-7-mut), luciferase activity was slightly, but not significantly increased in comparison with *HMGA2*-WT (WT, ctr), independent of the IMP3 expression status.

To confirm that the observed regulatory effects on *HMGA2* expression are in fact due to changes in IMP3-binding affinity, we performed quantitative EMSAs (Fig. 8d). Whereas IMP3 binding to the let-7-mut sequence was nearly unaffected compared with WT *HMGA2*, the affinities for IMP3-mut and IMP3-let-7-mut were decreased 2.5- to 3.5-fold, supporting the activities of our *HMGA2* luciferase constructs. Notably, with increasing concentrations (>60 nM), higher-order complexes could be detected, indicating multiple IMP3 molecules bound to this RNA.

Taken together, our in-depth analysis of sequence requirements for IMP3–RNA interaction and the functional validation supports the suggested safe-housing mechanism: Through sequence-specific formation of RNP complexes, IMP3 shields a specific region within the *HMGA2* 3′-UTR that contains miRNA-binding sites in close proximity, thereby protecting the mRNA from let-7-mediated repression.

## Discussion

Members of the IMP protein family are prime examples for multidomain RBPs, where both affinity and specificity are achieved through simultaneous engagement of multiple domains with their respective RNA elements. Although bioinformatic analyses can predict some features of RNA recognition by multidomain proteins^26,28^, systematic experimental approaches to study combinatorial RNA recognition by multidomain RNA-binding proteins have not been reported so far. Also, commonly employed global approaches to map protein–RNA interactions, such as CLIP, RIP, and RNACompete, have been analyzed with the aim to reveal short consensus sequences. Thereby, the systematic description of multidomain RBPs as well as rational searches for high-confidence and functional target sequences were severely limited^20^.

Here, we focused on IMP3 to dissect its complex RNA binding through a systematic SELEX-seq approach: We found that all di-domains (RRM1–2, KH1–2, and KH3–4) were active in RNA binding, while most previous studies had argued that only the KH domains 3 and 4 guide RNA recognition^5,14–16,29,30^. Our SELEX approach based on a N_40_-degenerate sequence revealed that the KH domains recognize two different types of RNA motifs: CA-rich motifs and elements with a common GGC core. We note that choosing 40 degenerate positions for our SELEX analysis limits the analysis of motif combinations, yet allows the enrichment and spacing analysis for at least two adjacent motifs, so that combined with the parallel study of various subdomains, a model for RNA binding of full-length IMP3 with its six RNA-binding modules that span ~100 nts could be derived. Structural analysis of the KH1–2 and RRM1–2 tandem domains and mapping of RNA interactions by NMR corroborated the specific interaction between subdomains and SELEX-derived RNA motifs. ITC clearly proved a cooperative interaction of tandem KH1–2 with a properly spaced, bipartite RNA motif. Our data suggest that in complex with KH1–2—similar to the situation with KH3–4— the RNA adopts a looped conformation that fits the narrow window for linker length between motifs.

In contrast to the IMP1-associated CGGAC motif, we find that IMP3 recognizes two related GGC-core elements (GGCA and CGGC), including their relative arrangement in combination with an additional CA-rich motif. Therefore, our data argue for KH1–2 and KH3–4 acting as independent tandems, both recognizing a combination of one CA-rich motif and one GGC element, with KH1 and KH4 binding the respective GGC elements.

Specifically, for KH3–4, and to a lesser extent for KH1–2 and KH1–4, we also observed an enrichment of AU-rich sequences. However, these sequences were underrepresented in full-length IMP3. This may reflect unspecific binding caused by protein truncation. Indeed, C-terminally shortened variants of KH3–4 and KH1–4 were diminished in RNA binding. To experimentally test the contribution of AU-rich sequences to IMP3–RNA binding, we quantitatively assayed RNA binding of full-length IMP3 protein to an additional mutant derivative of the 101-mer RNA, CA->UA, where both central CA elements were changed to AU (see Supplementary Fig. 3b): RNA-binding affinity of the 101-mer_CA->UA mutant RNA was reduced to about 18-fold (as seen also for the allUG mutant), so that we conclude that AU elements cannot functionally replace the CA elements in RNA binding.

In contrast to all previous reports^5,29,30^, we found that the N-terminal RRMs also contribute to RNA binding. The analysis of spacing between motifs revealed that all CA-rich motif combinations, but not combinations with the GGC-core elements, were highly enriched in each individual position within the 25-nts window. Most probably, this reflects a specificity for extended CA-repeat elements and binding of several RRM1–2 molecules to CA-rich sequences within the same RNA during the SELEX process. The observed CA specificity was lost under the stringent washing conditions during SELEX rounds 3 and 4, indicating less robust interactions in comparison with the KH domains. However, our in vitro validation with an RRM1-mutated full-length IMP3 supports an active role of RRM1–2. Based on the conservation of the RNP motifs, we infer that only RRM1 actively contributes to binding, which is supported by our NMR-binding data. A model of the RRM1–2 tandem based on NMR and SAXS data suggests that the domains adopt a compact fold, where RRM2 is only indirectly involved in RNA binding, perhaps by stabilizing a compact RRM1–2 arrangement.

Based on these motif analyses with isolated di-domains, we designed a prototypic RNA-target sequence within a 101-nt RNA that integrates the five SELEX-derived motifs with appropriate spacing. This model was tested and validated by mutational analysis with the 101-mer RNA and in vitro binding of well-known IMP3-target mRNAs containing the SELEX-derived motif array (e.g., *ANKRD17*, *IGF2*, and *HMGA2*). In fact, the consensus sequence bound to IMP3 with a high affinity, depending on the presence of the individual sequence elements, and involving all tandem RBDs. Importantly, our consensus motif array also allowed the successful prediction of IMP3 mRNA targets (see Fig. 7), further corroborating the validity of our approach.

We observed that isolated tandem domains (e.g., KH3–4) seem to tolerate the enriched motif combinations in both possible arrangements, a phenomenon that was previously described for KH3–4 of IMP1^14,15^. In our spacing analysis, this effect was more pronounced for KH3–4 and KH1–4 in comparison with KH1–2 alone. However, the NMR data of KH1–2 with a corresponding 34-mer RNA ligand indicate a certain degree of dynamic binding judged from the differential line broadening. The dynamic binding could involve the recognition of the 34-mer RNA in both orientations, i.e., with distinct looping of the RNA by the KH tandem domain. However, we cannot exclude unspecific higher-order oligomers at concentrations of NMR experiments, where line broadening is fostered by the formation of RNA–protein complexes with stoichiometries ≥2:1 that are in exchange with the 1:1 complex. Interestingly, a preference for one orientation (GGC-CA or CA-GGC) was detected for KH1–2 within the full-length IMP3 protein, indicating a restricted flexibility of the domains in their canonical context. This is further reflected by a decreased affinity when the order of KH1–2 RNA elements is swapped within the 101-mer RNA. The proposed topology of RNA elements and RBDs may be induced and stabilized additionally by the kinetic rates of binding, as suggested by Ramos and co-workers for looped RNA around KH3–4 at in vivo concentrations^16^. Notably, the NMR data of KH1–2 with a corresponding 34-mer RNA ligand show significant line broadening that is primarily caused by the increase in molecular weight.

In summary, we provide the first domain-resolved insight into the complex process of IMP3–RNA recognition through concerted interaction of multiple, clustered RNA sequence elements and all RBDs of IMP3. Multivalent interactions of individual domains, each with limited specificity, cooperatively add up to the very specific engagement of full-length protein with target RNAs^22,30^. This greatly exceeds previous studies, including large-scale surveys of many RNA-binding proteins^26,28^, which for the most part were restricted to short recognition sequences. These may even be misleading in many cases, since only particularly dominant sequence elements are usually identified by these approaches. Considering that most RBPs belong to the multidomain type^21,36,37^, our approach presented here on the IMP3 example should advance our understanding of clustered target RNAs^38–41^, and should help in global rational searches for functional target sites as well as in future engineering of tailored multidomain RBPs^42^.

## Methods

### Protein expression and purification

The full-length (FL) and truncated IMP3 derivatives used for SELEX experiments were ordered as codon-optimized DNA fragments encoding FL-IMP3 (Met1–Lys579), RRM1–2 (Met1–Asn163), KH1–2 (Pro164–Phe376), KH3–4 (Pro377–Lys579), and KH1–4 (Pro164–Lys579) (ThermoFisher), with additional His_6_-tag and TEV-cleavage site, and were cloned into the pGEX-6P2 expression vector (GE Healthcare). The GST–IMP3–TEV–His fusion proteins were purified via Ni^2+^-agarose beads (Qiagen), and the His_6_-tag was subsequently cleaved off through TEV protease according to the manufacturer’s instructions (Life Technologies). All protein preparations were dialyzed (20 mM Tris-HCl, pH 7.8, 20% glycerol, 100 mM KCl, 0.2 mM EDTA, and 1 mM DTT) and stored at −80 °C. IMP3 RNA-binding domain mutants were produced by PCR mutagenesis, using the Q5 Site-Directed Mutagenesis Kit following the manufacturer's instructions (NEB).

For structural studies, RRM1–2 (Lys2–Asp156) and KH1–2 (Lys192–Ile355) tandem-domain expression constructs were cloned from the human IMP3 full-length protein sequence optimized for expression in *E. coli*. The Δ versions of KH1–2^43^ were created by restriction-free site-directed mutagenesis. Proteins were expressed as thioredoxin fusion proteins comprising an N-terminal His_6_-tag and a TEV-cleavage site between thioredoxin and the gene of interest in the pETTrx1a vector (obtained from Gunter Stier, EMBL, Heidelberg). RRM1–2 was expressed by inoculating an LB overnight culture with a clone from a freshly prepared BL21 (DE3) LB culture plate supplemented with 0.35 mg/ml kanamycin. The culture was diluted into the medium of interest and grown to an OD_600_ of ~0.8 before induction with 0.5 mM IPTG. Cells were then grown for another 4–6 h at 37 °C before harvesting. Pellets were resuspended in lysis buffer (50 mM Tris, 300 mM NaCl, 4 mM TCEP, 15 mM imidazole, 1 mg/ml lysozyme, 10 µg/ml DNase I, and protease inhibitors, pH 8.0), incubated on ice for 30 min, and sonicated. Cleared lysates were subjected to Ni^2+^-agarose beads. After intensive washing, beads were incubated with 500 µg/l culture of TEV protease in lysis buffer for 3 h with gentle shaking at room temperature. Subsequently, the bead supernatant was collected, concentrated, and gel-filtrated in 20 mM Bis-Tris, 500 mM NaCl, and 2 mM TCEP, pH 6.5. The respective protein–monomer peak was pooled and salt concentration was adjusted to 150 mM. For RRM1–2, we included an additional ion-exchange chromatography step to reduce the level of nucleic acid contaminations. This was carried out on a 5/5 MonoS cation exchange column (GE Healthcare), running a gradient from 50 to 1000 mM sodium chloride in 20 mM Bis-Tris and 2 mM TCEP, pH 6.5. Fractions of intact protein were pooled and dialyzed against the final buffer as before.

### SELEX (systematic evolution of ligands by exponential enrichment)

The RNA pool with a degenerate sequence of 40 nucleotides (N_40_) was prepared by T7 transcription. In total, 40 pmol of full-length GST–IMP3 truncated derivatives, or GST alone (as negative control), were used for four rounds of selection with 4 nmol of SLX-N_40_ transcript. The stringency of washing steps (10 mM Tris-HCl, pH 7.5, 100/300/600 mM KCl, 2.5 mM MgCl_2_, and 0.1% Triton X-100) was increased for each round of selection (R1: 3 × 100 mM; R2: 2 × 100 mM, 1 × 300 mM; R3: 1 × 100 mM, 2 × 300 mM; R4: 1 × 300 mM and 2 × 600 mM KCl washing buffer). SELEX selections were carried out with the fusion proteins bound to glutathione–sepharose (GE Healthcare). RNA aliquots from each round were used for barcoding by reverse transcription with the SLX_RX reverse primers. cDNA libraries were amplified by PCR (17 cycles; SLX_Sol-5xN_fwd and SLX_Sol_rev). The final library pool was subjected to high-throughput sequencing on a MiSeq platform (single-read 150 bp, Illumina). PhiX control library was added to increase sample complexity (Illumina). For primer sequences, see Supplementary Data 3. Note that GST tags are known to form stable dimers. Our analysis of GST-tagged FL-IMP3 suggests that this dimerization does not affect the observed RNA interactions (Supplementary Fig. 4); however, potential effects of dimer formation on other GST-tagged constructs have not been ruled out.

### SELEX-seq data analysis

To identify the enriched binding motifs, sequence reads were first sample-barcode sorted, trimmed by PCR primer sequences on both ends, and further random-barcode filtered to obtain 38- to 40-nt sequence tags of the RNA pools for each sample or round (numbers of sequence tags given in Supplementary Fig. 1). The numbers of sequence tags (from each SELEX sample/round) containing either one of the 256, 1024, or 4096 possible tetramer, pentamer, or hexamer motifs, respectively, were summarized, and the *z*-score values were calculated for enrichment of each motif (Supplementary Data 1). Each SELEX sample/round was normalized to the corresponding GST SELEX rounds (as a negative control and for background correction).

For spacing analysis, sequence tags (round 4 for full-length IMP3, KH1–2, KH3–4, and KH1–4; and round 2 for RRM1–2) containing two tetramers with a spacing of 0–25 nts were summed up, and the *z*-score values were assigned. For each of the 65,536 possible combinations of two tetramers, the *z*-score mean values for spacing of 0–25 nts were determined for enrichment ranking. Among the top-500 enriched tetramer combinations identified for full-length IMP3, the following were selected and grouped (see Supplementary Data 2):

- Top-10 most-enriched combinations of two CA-rich sequences.
- CA-rich sequence on the 5′ end and GGCA element 3′.
- GGCA element on the 5′ end and CA-rich sequence 3′.
- CA-rich sequence on the 5′ end and CGGC element 3′.
- CGGC element on the 5′ end and CA-rich sequence 3′.
- Two GGC-core elements.

CA-rich refers to tetramers containing at least three C or A residues, alternating at least once, and excluding CCC, AAA, and any G nucleotides.

For each group, the *z*-score mean values for individual positions (0–25 nts) were assigned and represented as a heatmap in Fig. 2a (top panel). The motif combinations obtained from (b) to (e) were subsequently used for spacing analysis of the truncated KH-domain-containing derivatives (KH1–2, KH3–4, and KH1–4; bottom panels). For RRM1–2, and in addition to spacing information of CA-rich sequences from (a), motif combinations obtained from (b) and (d) (5′-GGC-CA-3′), as well as (c) and (e) (5′-CA-GGC-3′), were combined and presented in a summarized format (middle panel).

### Correlation of IMP3 iCLIP and SELEX-motif array occurrence

Sequencing data for the IMP3 iCLIP in HepG2^27^ are available from the Sequence Read Archive (SRA) of NCBI (SRP139915).

The annotated 3′-UTRs (Comprehensive Gene Annotation Set from GENCODE version 19) were selected to evaluate IMP3 in vivo RNA-binding efficiency, with HepG2 whole-cell polyA+RNA-seq data from ENCODE/CSHL (genome.ucsc.edu) applied as a RNA expression reference. A binding index (B index) of each in HepG2 cells expressed 3′-UTR was calculated as the ratio of iCLIP-tag counts and the expression level estimated by RNA-seq read coverage.

To identify SELEX-derived motif arrays, 3′-UTR sequences were screened by sequentially applying the following criteria (note that some of these spacing constraints were set more relaxed than in our in vitro derived model (Fig. 2b)), allowing for longer RNA-structural loops:

- Two flanking GGC-core elements (CGGC or GGCA), with a spacing of 30–200 nt, (GGC) - N_30–200_ - (GGC).
- Two CA elements, out of the 13 tetramer CA elements used for spacing analysis of 4-mer motif combinations (Supplementary Data 2), with a minimum spacing of 6 nt between them and at least 10 nt on either side toward the two flanking GGC-core elements, (GGC) - N_≥10_ - (CA) - N_≥6_ - (CA) - N_≥10_ - (GGC).
- Downstream or upstream of the two flanking GGC-core elements, a CA motif within a distance of 6–200 nt, (GGC) - N_6–200_ - (CA) or (CA) - N_6–200_ - (GGC).

### Commercial RNAs

The RRM1–2-related RNAs (CA)_5_, (CA)_3_, (GU)_5_, the KH1–2-related GGC and CA 7-mers, and the 34-mer were obtained from IBA (Göttingen) or Eurofins (Ebersberg). Lyophilized RNAs were dissolved in nuclease-free water, heated to 95 °C for 5 min, snap-cooled, aliquoted, and stored at −80 °C.

### Crystallization, diffraction data collection, and processing

The crystallization experiments for IMP3 KH1–Δ2 domain were performed at the X-ray Crystallography Platform at Helmholtz Zentrum München. Initial screening was done at 292 K, using 12 mg/ml of protein with a nanodrop dispenser in sitting-drop 96-well plates and commercial screens. Crystals appeared after 1–2 days with sufficient size for X-ray diffraction experiments. The best dataset was collected for a crystal grown in 0.08 M magnesium acetate, 0.05 M sodium cacodylate, pH 6.5, and 30% w/v polyethylene glycol 4000 (Hampton Research NATRIX screen). For the X-ray diffraction experiments, the crystals were mounted in a nylon fiber loop and flash-cooled to 100 K in liquid nitrogen. Prior to freezing, the crystals were protected with 25% (v/v) ethylene glycol. Diffraction data were collected at 100 K on the PX X06SA beamline (SLS, Villigen). The diffraction data were indexed and integrated using *XDS*^44^ and scaled using *SCALA*^45^. Intensities were converted into structure-factor amplitudes using the program *TRUNCATE*^46^. Supplementary Table 1 summarizes data collection and processing statistics.

### Structure determination and refinement

The structure of KH1–2 domains was solved by the *Auto-Rickshaw* pipeline^47^. Three-dimensional model of KH1–2 domains of the neuronal splicing factor Nova-1 (PDB-ID: 2ann)^48,49^ was used as a search model. For the molecular replacement step followed by several cycles of automated model building and refinement, the *Auto-Rickshaw* pipeline involved the following X-ray crystallography software: MORDA, *CCP4*^50^, *SHELXE*^51^, *BUCCANEER*^52^, *RESOLVE*^53^, *REFMAC5*^54^, and *PHENIX*^55^. Model rebuilding was performed in *COOT*^56^. The further refinement was done in *REFMAC5*^54^ using the maximum-likelihood target function. The stereochemical analysis of the final model was done in *PROCHECK*^57^ and *MolProbity*^58^. The final model is characterized by R/R_free_ factors of 23.29/29.27% (Supplementary Table 1). Atomic coordinates and structure factors have been deposited in the Protein Data Bank under accession code 6GQE.

### NMR spectroscopy

For NMR measurements, proteins were expressed in M9 media supplemented with 0.5 mg/ml ^15^N ammonium chloride (titrations and relaxation experiments) and 2 mg/ml ^13^C glucose (triple-resonance experiments for backbone assignments). Wild-type KH1–2 has additionally been expressed in 99.5% D_2_O, following a previously described protocol^59^. Briefly, cells were grown in sequential steps of 0% D_2_O and 2 g/l glucose (50 ml), 50% D_2_O and 2 g/l glucose (250 ml), and finally 99.5% D_2_O and 2 g/l ^2^H;^13^C glucose (2 l) with full transfer of cell mass between steps. All experiments were performed in 20 mM Bis-Tris, 150 mM NaCl, 2 mM TCEP, 0.02% sodium azide, and 5–10% of D_2_O. NMR backbone assignments have been obtained using the following experiments: HNCA, HNcoCA, HNCACB, CBCAcoNH, HNCO, HNcaCO, and ^15^N-edited NOESYs. All datasets were acquired from Bruker Avance spectrometers of 600–950-MHz proton frequency equipped with triple-resonance cryo-probes using *Topspin 3*.*2*. The data were processed with *Topspin* and analyzed using the *CCPNMR Analysis* software package^60^ and *SPARKY*^61^. Sample concentrations were 250–650 µM.

RRM1–2 ^15^N autorelaxation and {^1^H}–^15^N heteronuclear NOE data were recorded with a 300 µM sample using pseudo-3D experiments with the delays 4, 8, 16, 32, 48, 64, 96, 128, 192, 256, 512, and 1024 ms for *R*_1_ and the delays 5, 10, 20, 40, 60, 80, 100, 125, 150, 200, and 300 ms for *R*_2_. Peak intensities were fitted and plotted with *Analysis*. τ_C_ was calculated based on the ratio of *R*_1_ and *R*_2_. NMR titrations of KH1–2 versions and RRM1–2 were performed with samples of 50–100 µM protein by adding the denoted stoichiometries of RNA from a 4 mM stock solution. Combined chemical shift perturbations were calculated using the formula Δδ = [6(Δ^1^H)^2^ + (Δ^15^N)^2^]^0.5^. All NMR experiments were carried out at 25 °C.

### Static-light scattering (SLS)

SLS runs were performed on a Malvern *Omnisec* device with an integrated sample changer and equipped with a semianalytical SD200 10/300 Superdex column (GE). Samples of RRM1–2 had concentrations as indicated; the used sample volume was 125 µl. Runs were performed in buffers as for NMR, but no D_2_O. UV (260 and 280 nm), right-angle light-scattering and refractive index data were analyzed using the integrated *Omnisec* software, and molecular weights were determined using a *dn/dc* value of 0.185 for protein. Therefore, peak picking and baseline definition were performed automatically or manually. The system was calibrated with 5 mg/ml bovine serum albumin (66.5 kDa) as a standard.

### Small-angle X-ray scattering (SAXS)

SAXS experiments were performed in-house or on beamline BM29 at ESRF, Grenoble, France. Sample concentrations were 1–7 mg/ml. Reference runs in buffers were performed multiple times and used for buffer subtractions. Measurements were carried out as technical triplicates in four to ten frames to enable the exclusion of data in the case of radiation damage. Data were processed and analyzed with the ATSAS^62^ package version 2.8, including the plot of paired-distance distribution, P(*r*), the determination of *D*_max_ and *R*_g_, and the calculation of Porod volumes and molecular weights with *DATPOROD*. Theoretical scattering curves derived from the KH1–2 crystal structure or RRM1–2 models were calculated with *Crysol*^63^.

### RRM1–2 modeling

Due to the lack of an experimental structure of RRM1–2, we used SAXS data to filter randomized tandem arrangements. Therefore, RRM1 was modeled based on the IMP2 RRM1 NMR structure (PDB-ID: 2cqh), including residues 1–72. For the RRM2, we used the available structure (PDB-ID: 2e44) and adjusted the domain boundaries to residues 80–156. This fragment was in perfect fit with a CS-Rosetta-based structure based on our backbone NMR data. The linker region 73–79 was kept flexible and the two domains used as an ensemble in 10,000 random starting structures were generated with *EOM2*^64^ and fitted against the SAXS scattering curve at the highest concentration. We obtained an ensemble of four structures with populations of 60, 20, and two times 10% that showed a *χ*^2^ fit of 1.335. We chose the highest-populated structure, that also represented the most compact moiety (*D*_max_ of 61 Å) and used it to include the following restraints: The 7-mer linker (residues 73–79) was rationally probed for possible conformations, i.e., the minimum distance between residues 72 and 80 in a U-turn loop (6 Å), within a α-helix (12 Å) or the maximum distance when arranged in a β-strand (26 Å). The first would have led to steric clashes between RRM1 and 2, and since our secondary chemical shift data did not reveal a clear preference for α-helical or β-strand elements, we set the distance to be 16 Å. That allows for sufficient flexibility but would still be in line with a high degree of rigidity (see heteronuclear NOE and relaxation data) and fulfills the obtained *D*_max_ of 54 Å when manually arranging RRM1 and 2. In order to satisfy CSPs, we included a maximum distance of 30 Å between residues Val35 (central in RNP2 of RRM1) and Ser127 (RRM2). The latter—despite nonfunctional RNPs in RRM2—still significantly senses the binding of (CA)_3_ RNA, which would approximately comprise a maximum extension of 30 Å. Finally, the relative twist of RRM1 versus RRM2 around the positively charged inter-domain linker was limited, given the fact that it senses strong CSPs (see Lys77), indicating that it could be arranged along with the RNA. As such, we decided to prevent a cross-brace possibility for a linker and RNA and suggest the RNA to bind along the RRM1 β-sheet and the linker, thereby indirectly interacting with Ser 127/128. Hence, we put a 15-Å distance between the strongly shifting residue Thr115 and Glu55 to impair the free rotation of domains. All two-domain models were used in the program *Coral*^65^ and fitted against the scattering curves, until the crucial parameters *D*_max_, *R*_G_, and Porod volume were optimized and the model was approximately in line with the CSP plot. The final model showed a *χ*^2^ of 1.9, as given in Supplementary Fig. 8. Note that the linker is not part of the model.

### Isothermal titration calorimetry (ITC)

ITC measurements were performed with a MicroCal PEAQ-ITC device (Malvern, United Kingdom) in the NMR buffer. In all experiments, RNA was titrated from a stock of 10–20-fold concentration excess to 20–40 µM protein provided in the reaction cell. In a standard ITC run, we used 19 injections of 2 µl with 150-s spacing at room temperature with a 750-rpm stirring speed. Raw data were analyzed with the integrated analysis tool and heat production was fitted to a one-site binding model. Where appropriate we performed a buffer subtraction.

### Electrophoretic mobility shift assay (EMSA)

RNAs of the 101-mer series were produced and ^32^P-UTP-labeled by T7 transcription from annealed oligo cassettes. SELEX-motif-containing regions of *IGF2* (NM_001007139.5), *HMGA2* (NM_003483.4), and *ANKRD17* (NM_032217.4) transcripts were PCR amplified and used for T7 transcription and labeling (sequences given in Supplementary Data 3). Binding reactions were performed in binding buffer (10 mM Tris-HCl, pH 7.5, 150 mM NaCl, 0.5 mM EDTA, 0.5 mM DTT, 0.1% NP-40, 5% glycerol, supplemented with RNaseOUT, as well as tRNA and BSA as nonspecific competitors) containing the purified protein (titrations from 0 to 40, 0 to 80, 0 to 120, 0 to 160, or 0 to 320 nM) and the ^32^P-UTP-labeled RNA (5 nM) in a final volume of 10 µl. The reaction was first incubated for 30 min at room temperature, and then placed on ice for 5 min. Each sample was supplemented with loading buffer (1x TBE, 0.05% bromophenol blue), and loaded onto a cold native 5% TBE gel (containing 5% glycerol) that had been pre-run for 30 min. Electrophoresis was performed for 50 min with 45 mA at 4 °C. Complexed and free RNA was visualized for quantitation by the Typhoon FLA 9500 Phosphorimager system (GE Healthcare). Curve fitting of raw data using the quadratic binding equation^66^ or the Hill equation (${y} = V_{{\mathrm{max}}}\frac{{x^n}}{{k^n + x^n}}$), and *K*_D_ calculations from experimental replicates were performed with OriginPro (OriginLab). Whenever source data are available, this is indicated within the respective figure legends.

### IMP3 pulldown with biotinylated RNAs and western blot

RNAs of the 101-mer series were produced by T7 transcription (T7 High-Yield Kit, NEB) from annealed oligo cassettes and chemically modified by 3′-biotinylation^67^. For pulldown of IMP3 from HeLa cell lysate, 2.5 × 10^6^ cells were lysed in lysis buffer (50 mM Tris-HCl, pH 7.4, 150 mM NaCl, 5 mM EDTA, 1% NP-40, and 0.1% SDS) and incubated with 40 pmol of 3′-biotinylated RNA bound to NeutrAvidin agarose beads (ThermoFisher) in a total volume of 200 µl for 30 min at room temperature. Pulldown of recombinant IMP3 was performed by incubation of 10 pmol 3′-biotinylated RNA bound to NeutrAvidin agarose beads (ThermoFisher) with 1 pmol protein in binding buffer (10 mM Tris-HCl, pH 7.4, 100 mM KCl, 2.5 mM MgCl_2_ and 0.1% Triton X-100) in a total volume of 200 µl for 30 min at room temperature. After three washing steps with washing buffer (1x WB100, 2x WB300 for pulldown from lysate, and 1x WB100, 2x WB600 for recombinant protein; 10 mM Tris-HCl, pH 7.4, 100–600 mM KCl, 2.5 mM MgCl_2_, and 0.1% Triton X-100), bound protein was released in SDS-sample buffer (50 mM Tris-HCl, pH 6.8, 2% SDS, 10% glycerol, 2.5% 2-mercaptoethanol, and 0.05% bromophenol blue) and heat denaturation at 95 °C for 10 min. Samples (10% input and 50% pulldown) were analyzed by SDS-polyacrylamide gel electrophoresis (10% polyacrylamide gel) and Western blotting with polyclonal anti-IMP3 antibody (Millipore) against endogenous IMP3, or anti-GST antibody (Pharmacia Biotech) against the recombinant and GST-tagged IMP3 version.

### RNA immunoprecipitation and qPCR (RIP-qPCR)

Cell lysates were prepared in RIPA buffer (50 mM Tris-HCl, pH 7.4, 150 mM NaCl, 5 mM EDTA, 1% NP-40, and 0.1% SDS). Antibody binding was performed for 2 h at 4 °C, using a polyclonal anti-IMP3 antibody (Millipore) and as a mock control, anti-FLAG antibody (Sigma-Aldrich). Bead capturing was carried out for 1 h at 4 °C with protein-G dynabeads (Life Technologies), and protein–RNA complexes were washed (50 mM Tris-HCl, pH 7.4, 150/300/600 mM NaCl, and 0.05% Tween-20), increasing the stringency during the washing steps. RNA from the input and from the immunoprecipitated fractions was extracted by TRIzol (Ambion), followed by ethanol precipitation and reverse transcription (qScript cDNA Synthesis Kit, Quanta). Real-time PCR was carried out using Luna Universal qPCR Master Mix (NEB) and an Eppendorf realplex thermocycler. The fraction of bound target RNAs in RIP assays was calculated with each target normalized to the corresponding input fraction (results represented as percent of the input). Biological triplicates were used to calculate standard deviations. For primer sequences, see Supplementary Data 3.

### CRISPR/Cas9 genomic IMP3 knockout

For the CRISPR/Cas9-mediated genomic deletion of IMP3, ES-2 cells were transfected with two CRISPR guide RNAs (psg_RFP_IMP3_1, psg_RFP_IMP3_2) and Cas9 nuclease (pcDNA_Cas9_T2A_GFP), using Lipofectamine2000 (ThermoFisher) according to the manufacturer’s protocol. Single-cell clones were generated by seeding one RFP- and GFP-positive cell per well using flow cytometry (BD FACSAria II). The deletion of IMP3 was validated by Western blotting using paralog-specific anti-IMP3 antibodies (C-terminal clone 6G8, BSBS AB facility; N-terminal RN009P, MBL). CRISPR guide RNAs are summarized in Supplementary Data 3.

### Luciferase assays

 The region of the *HMGA2* 3′-UTR (NM_003483.4) containing the IMP3 SELEX-derived motif and the let-7 seed sequences, together with the respective mutants (IMP3-mut, let-7-mut, and IMP3-let-7-mut), were ordered as DNA fragments (Supplementary Data 3, ThermoFisher) and cloned into the pmirGLO-Dual-Luciferase miRNA Target Expression Vector (Promega). For luciferase reporter assays, 1.5 × 10^5^ ES-2 cells (with or without genomic IMP3-KO) were seeded per well, in a 12-well plate. Cells were transfected with 250 ng of plasmid DNA and 4 µl of Turbofect (ThermoFisher), and incubated for 24 h. After three washing steps with 1x PBS (Gibco), cells were lysed in 250 µl of 1x Lysis-Juice (PJK) according to the manufacturer’s instructions. Luminescence was monitored for Firefly luciferase, using the Beetle-Juice Kit, and for Renilla luciferase, using the Renilla-Juice Kit (both PJK) with a Centro LB 960 Luminometer (Berthold Technologies). Relative luciferase activities were calculated as a ratio of Firefly and Renilla raw values with three technical replicates per sample and a total of three independent biological replicates. ES-2 cells with or without IMP3-KO were characterized by SDS-polyacrylamide gel electrophoresis (10% polyacrylamide gel) and western blotting with antibodies specific for IMP1 (clone 6A9, BSBS AB facility), IMP2 (clone 6A12, BSBS AB facility), IMP3 (Millipore), and GAPDH as a negative loading control (Sigma).

### Reporting summary

Further information on research design is available in the Nature Research Reporting Summary linked to this article.

## Supplementary information

Supplementary Information

Reporting Summary

Description of Additional Supplementary Information

Supplementary Data 1

Supplementary Data 2

Supplementary Data 3

Source Data

## Data Availability

Crystallography data (PDB-ID: 6GQE), NMR amide chemical shifts for RRM1–2, KH1–2, KHΔ1–2, and KH1–Δ2 (BMRB-IDs: 27813, 27815, 27827, and 27816), and iCLIP data (SRA-ID: SRP139915) have been deposited to their relevant publicly accessible repositories. The source data underlying Figs. 3b–c, 4e, 5g, 6b, 7c, and 8b–d and Supplementary Figs 3a–c, 4b–d, 8e, and 9 are provided as a Source Data file. All other relevant data are available from the authors upon request.
