# Supplementary material for: Combinatorial recognition of clustered RNA elements by the multidomain RNA-binding protein IMP3
Source: Nat Commun. 2019 May 22;10:2266. doi: 10.1038/s41467-019-09769-8 (PMC6531468; doi:10.1038/s41467-019-09769-8)
Supplement: Supplementary file 1 — Supplementary Information [file 41467_2019_9769_MOESM1_ESM.pdf]

# *Supplementary Material*

## Combinatorial recognition of clustered RNA elements by the multidomain RNA-binding protein IMP3

Tim Schneider[1,7], Lee-Hsueh Hung[1,7], Masood Aziz[2,3], Anna Wilmen[1], Stephanie Thaum[1], Jacqueline Wagner[2], Robert Janowski[3], Simon Müller[4], Silke Schreiner[1], Peter Friedhoff[1], Stefan Hüttelmaier[4], Dierk Niessing[3,5], Michael Sattler[2,3]*, Andreas Schlundt[2,3,6]*, and Albrecht Bindereif[1]*

[1] Institute of Biochemistry, Justus-Liebig-University of Giessen, 35392 Giessen, Germany
[2] Center for Integrated Protein Science Munich (CIPSM) at Department of Chemistry, Technical University of Munich (TUM), 85747 Garching, Germany
[3] Institute of Structural Biology, Helmholtz-Zentrum München, 85764 Neuherberg, Germany
[4] Institute of Molecular Medicine, Section for Molecular Cell Biology, Faculty of Medicine, Martin Luther University Halle-Wittenberg, 06120 Halle, Germany
[5] Institute of Pharmaceutical Biotechnology, Ulm University, 89081 Ulm, Germany
[6] Current address:
Institute for Molecular Biosciences and Center for Biomolecular Magnetic Resonance (BMRZ), Goethe-University Frankfurt, 60438 Frankfurt/M., Germany
[7] These authors contributed equally to this work.

* Correspondence should be addressed to:
A.B. (albrecht.bindereif@chemie.bio.uni-giessen.de).
M.S. (sattler@helmholtz-muenchen.de), or
A.S. (schlundt@bio.uni-frankfurt.de).

**Supplementary Material comprises nine Supplementary Figures and two Supplementary Tables.**
**Supplementary Data 1-3 are provided as a separate file.**

## Supplementary Figure 1

**a**

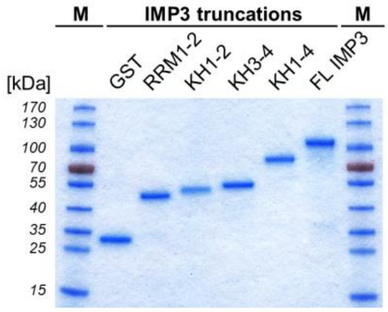


**b**

| N$_{40}$ SELEX-seq (rounds 1-4) | | | | | | | | |
|---|---|---|---|---|---|---|---|---|
| **variant** | **round of selection** | **reads** | **variant** | **round of selection** | **reads** | **variant** | **round of selection** | **reads** |
| **GST** | R1 | 307,964 | **RRM1-2** | R1 | 393,713 | **KH1-2** | R1 | 432,892 |
| | R2 | 268,041 | | R2 | 455,744 | | R2 | 469,040 |
| | R3 | 203,282 | | R3 | 473,852 | | R3 | 440,737 |
| | R4 | 270,665 | | R4 | 392,944 | | R4 | 398,455 |
| **KH3-4** | R1 | 403,880 | **KH1-4** | R1 | 444,630 | **FL IMP3** | R1 | 278,889 |
| | R2 | 392,764 | | R2 | 430,594 | | R2 | 252,757 |
| | R3 | 450,416 | | R3 | 389,832 | | R3 | 258,046 |
| | R4 | 409,200 | | R4 | 385,579 | | R4 | 255,152 |

**c**

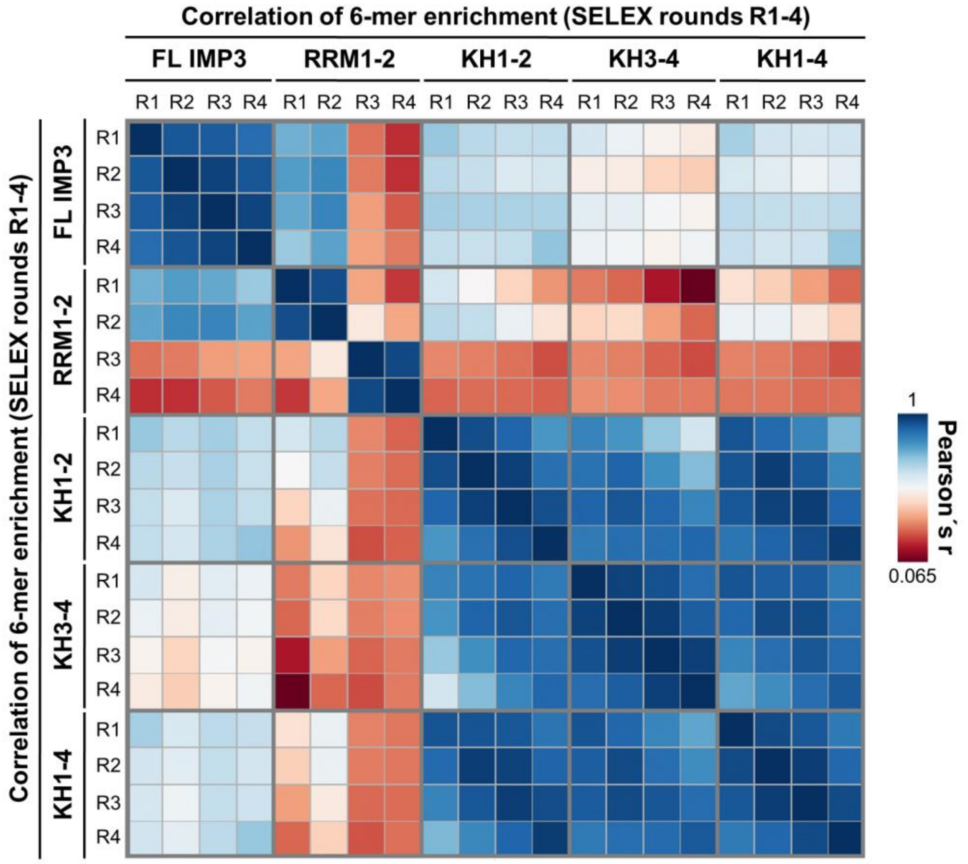

**Supplementary Figure 1.**
**SELEX-seq with truncated IMP3 derivatives (related to Fig. 1).**

(**a**) SDS-PAGE of purified, GST-tagged IMP3 truncation derivatives (as indicated), including full-length (FL) IMP3 (positive control) and GST alone (negative control). M, marker proteins (in kDa)
(**b**) Number of barcode-filtered sequence tags from SELEX-seq, as obtained for each round of selection (R1-R4) with the respective IMP3 truncation derivatives.
(**c**) Linear correlation (Pearson´s r) of 6-mer motif enrichment (shown as heat map) between full-length (FL) IMP3 and the truncation derivatives, shown for each SELEX round.

## Supplementary Figure 2

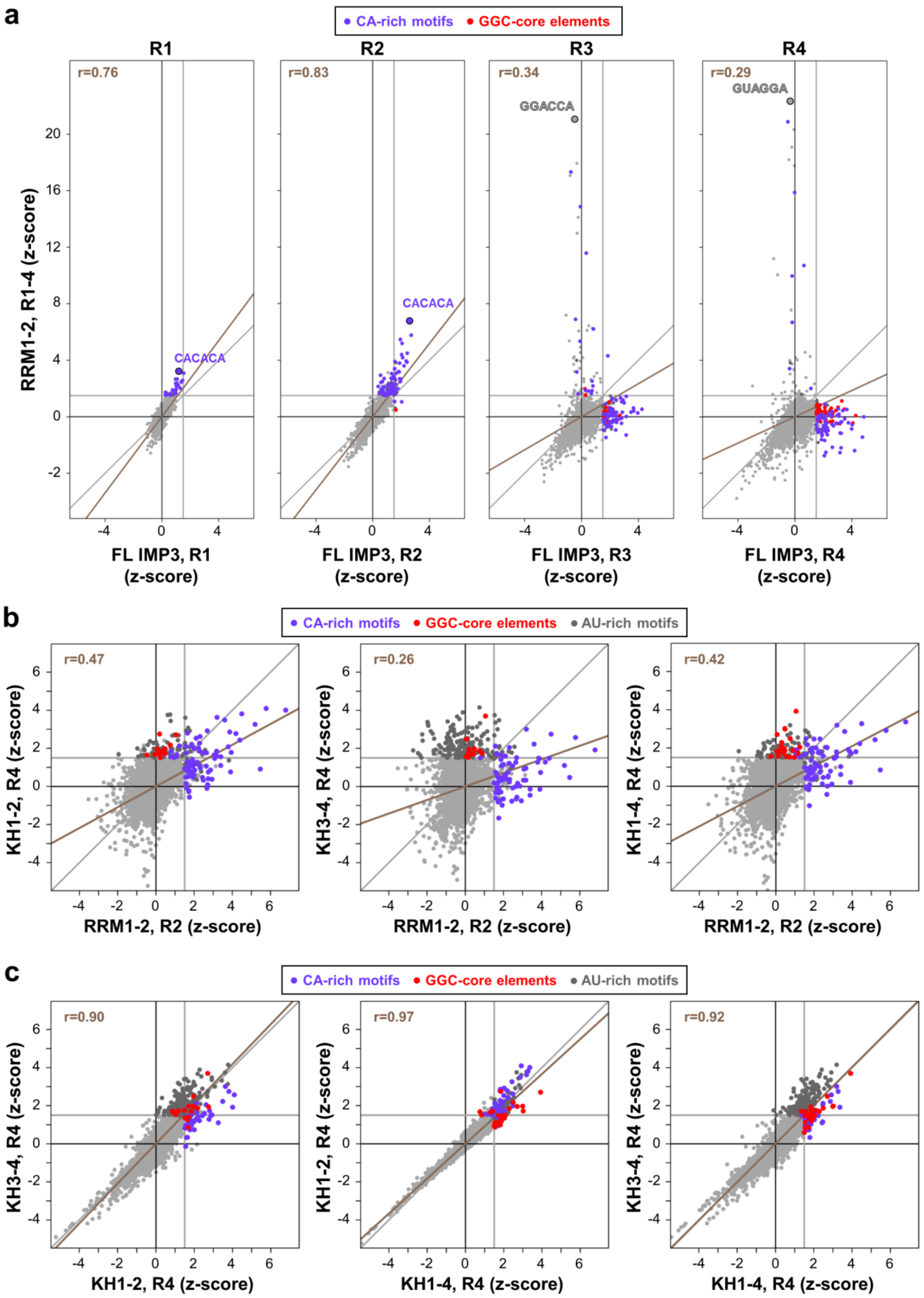

**Supplementary Figure 2.**
**Comparison of motif enrichment data for RRM1-2 and KH-containing domains (related to Fig. 1).**

(**a**) RRM1-2 loses CA-specificity during the third and fourth SELEX-selection. Correlation of 6-mer motif enrichment (measured by z-scores) for RRM1-2 (y-axis) in comparison to full-length IMP3 (x-axis) for each selection round (R1-R4). Motifs with z-scores higher than 1.5 (vertical/horizontal grey lines) in either x- or y-axis are highlighted in violet for CA-rich motifs, and in red for GGC-core elements. Pearson´s correlation by linear regression is shown as a brown line with correlation coefficients (r) indicated.
(**b**) RRM1-2 recognizes CA-rich sequences, but not the KH-specific GGC-core elements. Correlation of 6-mer motif enrichment for RRM1-2 (round 2, x-axis) in comparison to KH-containing domains (round 4, y-axis). Motifs with z-scores higher than 1.5 (vertical/horizontal grey lines) in either x- or y-axis are highlighted in violet for CA-rich motifs, in red for GGC-core elements, and in dark grey for AU-rich motifs.
(**c**) Correlation of 6-mer motif enrichment between KH-containing domains (round 4).

## Supplementary Figure 3

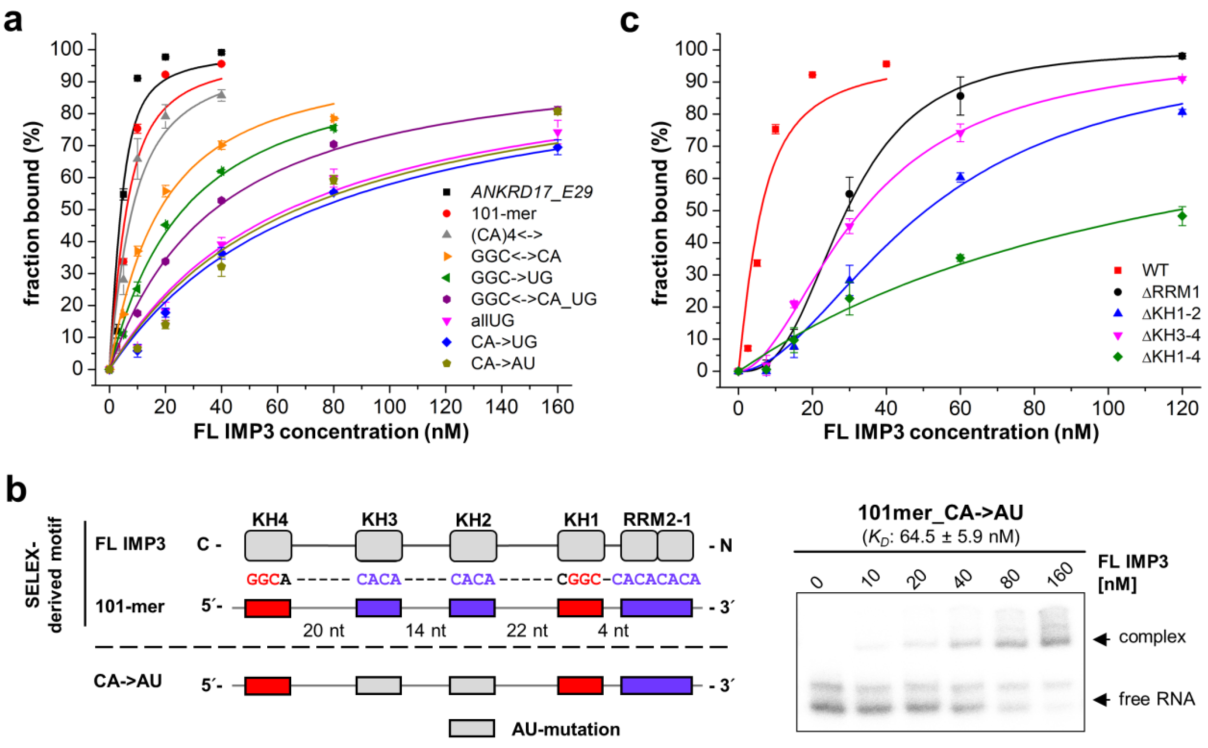


**Supplementary Figure 3.**
**Quantitative analysis of IMP3 binding to the 101-mer RNA (related to Figs. 3 and 6).**

(**a**) Binding curves for $K_D$-estimation of IMP3 interaction with RNAs of the 101-mer series as shown in **Fig. 3b** (mean and standard error of three EMSA experiments, data fitted with the quadratic binding equation).
(**b**) Contribution of AU-elements within the 101-mer RNA was tested by exchange of the central CA-motifs to AU (101-mer_CA->AU). Interaction of full-length (FL) IMP3 (0-160 nM) with this RNA was quantitated by EMSA (5 nM $^{32}$P-labeled RNA). For the corresponding binding curve for $K_D$-estimation see panel **a** (mean and standard error of three EMSA experiments)**.**
(**c**) Binding curves for $K_D$-estimation of wildtype IMP3 (WT, included for comparison, data fitted with the quadratic binding equation) and the protein mutants ΔRRM1, ΔKH1-2, ΔKH3-4 and ΔKH1-4 binding to the 101-mer RNA as shown in **Fig. 6b** (mean and standard error of three EMSA experiments, data fitted with the Hill equation, Hill coefficients of n = 2.8 ± 0.2 for ΔRRM1, n = 1.9 ± 0.1 for ΔKH1-2, n = 1.8 ± 0.1 for ΔKH3-4, and n = 1.0 ± 0.1 for ΔKH1-4).
Source data are provided as a Source Data file.

**Supplementary Figure 4**

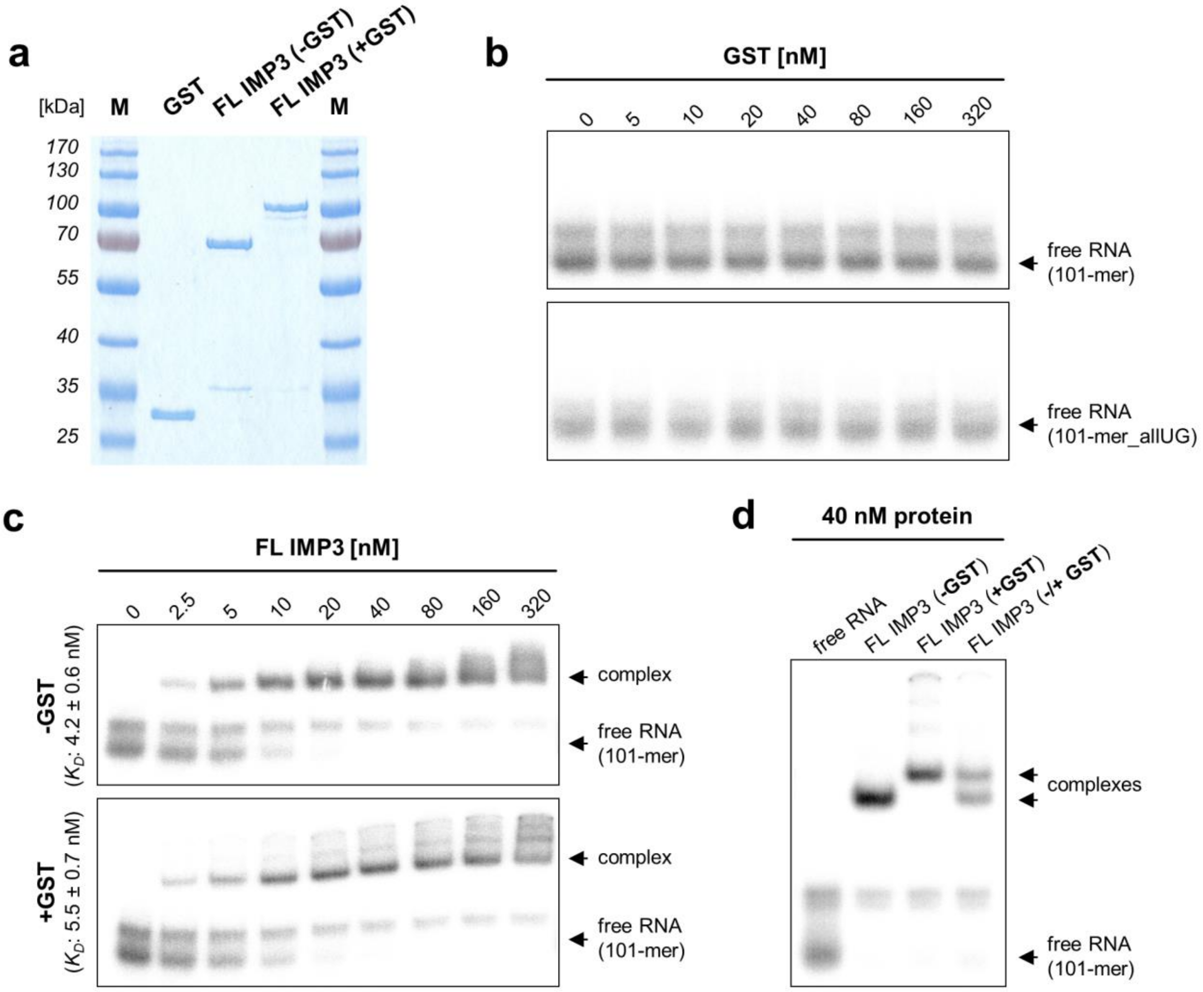


**Supplementary Figure 4.**
**Stoichiometry of IMP3 binding to the 101-mer RNA (related to Fig. 3).**

(**a**) SDS-PAGE analysis of purified GST protein, as well as full-length (FL) IMP3 derivatives with (+GST) and without GST-tag (-GST). M, marker proteins (in kDa).
(**b**) Electromobility shift assays (EMSA) of GST protein with $^{32}$P-labeled 101-mer RNA. GST protein (0-320 nM) was titrated to a constant concentration of RNA (5 nM, representative of two experiments shown).
(**c**) Interaction of full-length (FL) IMP3 derivatives (-/+GST) with the 101-mer RNA, assayed by EMSA. FL IMP3 derivatives (0-320 nM) were titrated to a constant concentration of $^{32}$P-labeled RNA (5 nM, $K_D$-estimations and standard errors of two experiments).
(**d**) Stoichiometry of IMP3-RNA complex, analyzed by EMSA. $^{32}$P-labeled 101-mer RNA (5 nM) was incubated with FL IMP3 protein without (-GST), FL IMP3 protein with GST-tag (+GST), or a 1:1 mixture of both derivatives (-/+GST), each at 40 nM protein, to assess potential formation of IMP3 heterodimer-RNA complexes (representative of two experiments shown).
Source data are provided as a Source Data file.

## Supplementary Figure 5

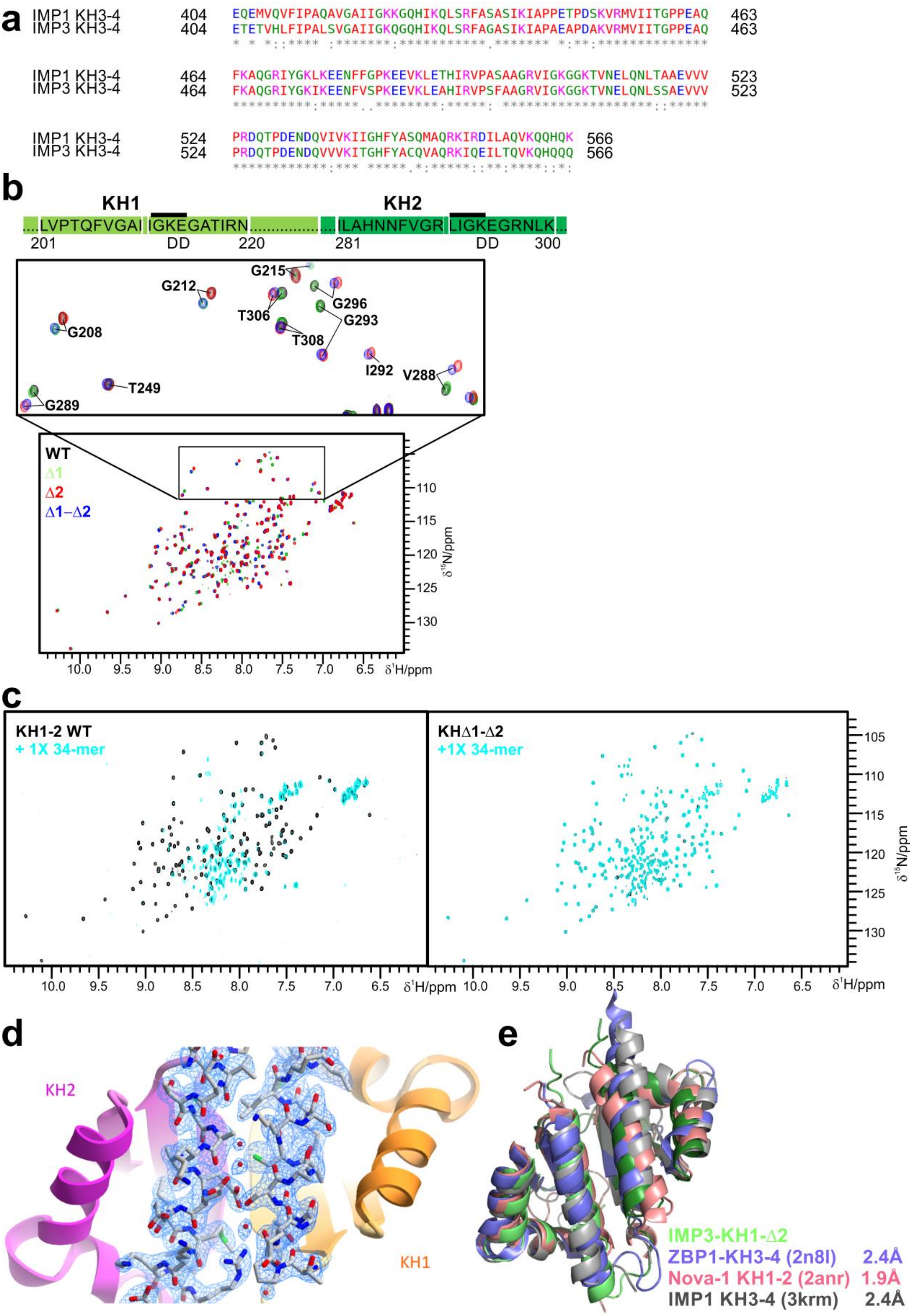

**Supplementary Figure 5.**
**Structure and RNA recognition by the IMP3 tandem KH1-2 domain (related to Fig. 4).**

(**a**) Sequence alignment of IMP1 and IMP3 KH3-4 tandem domains suggests similar RNA-binding preferences and modes.
(**b**) Proof of concept for GxxG-based mutational analysis of KH1-2 subdomains[43]. The top panel shows the GKEG regions of either KH domain with their adjacent amino acid sequence, zoomed into the respective region of interest in the full spectral overlay below. Positions of replacement with the two aspartates are indicated with 'DD'. Zoomed regions of $^{1}H$-$^{15}N$-HSQC overlays show all four versions of KH1-2 used in this study (see color code). The residues annotated serve as probes for a stretch of residues in KH1 and KH2. Where possible, both the wildtype and mutant form resonance positions are annotated.
(**c**) Left, $^{1}H$-$^{15}N$-HSQC overlay of wildtype KH1-2 alone and in presence of equimolar amounts of 34-mer RNA. Contours in the bound spectrum are not to scale to visualize broadened peaks close to the noise level. Right, $^{1}H$-$^{15}N$-HSQC overlay of the double knockout KH1-2 version Δ1-Δ2 alone and in presence of equimolar amounts of 34-mer RNA.
(**d**) Excerpt of the KH1-Δ2 crystal electron density map. The $2F_o$-$F_c$ map is contoured at 1σ and covers helices (shown as ball and stick) on the interface of the two KH domains. KH1 and KH2 domains are shown as orange and magenta ribbons, respectively. The figure was prepared using CueMol: Molecular Visualization Framework (http://www.cuemol.org/).
(**e**) Structural alignment of the KH1-Δ2 crystal structure with human IMP1 KH3-4[14], the chicken ZBP1 KH3-4[16] and the Nova-1 KH1-2[48] tandem domain structures. The latter was chosen as it represents the best hit in a DALI-based[49] search for similar structures. RMSD values are given.

**Supplementary Figure 6**

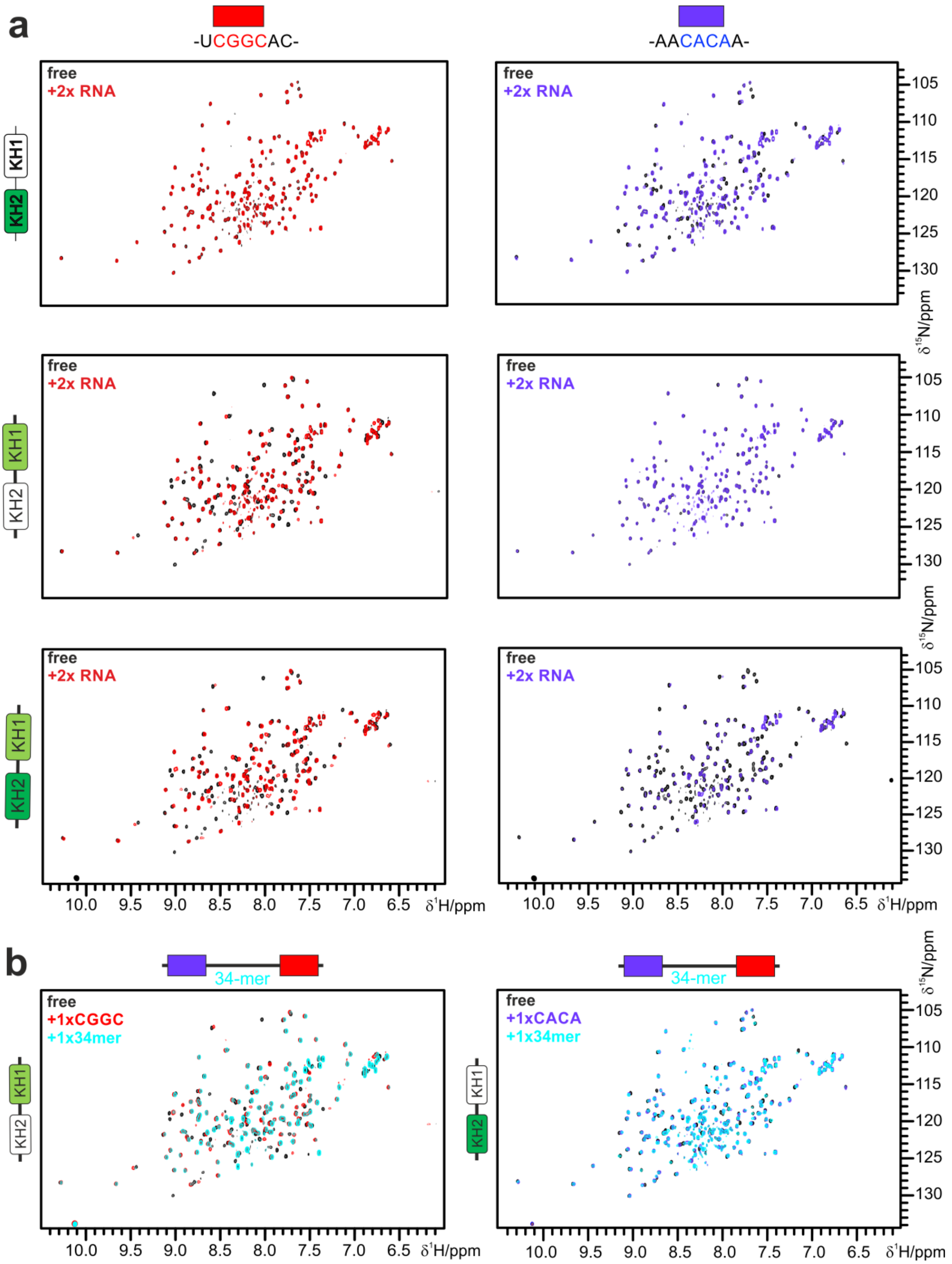

**Supplementary Figure 6.**
**Structure and RNA recognition by the IMP3 tandem KH1-2 domain (related to Fig. 4).**

(**a**) Full views of $^{1}H$-$^{15}N$-HSQC overlays shown in **Fig. 4d** with individual binding of 7-mer RNA motifs, UCGGCAC or AACACAA, to KH1-2 versions Δ1, Δ2 or the wildtype.
(**b**) Full views of $^{1}H$-$^{15}N$-HSQC overlays shown in **Fig. 4d** comparing binding of equimolar 34-mer RNA to KH1-2 versions Δ1 or Δ2 with binding of the short CGG- or CA-containing RNAs, respectively. Note that icons for protein domains and RNAs are shown in C-N and 5'-3' orientation for better comparison with **Fig. 4**, respectively.

**Supplementary Figure 7**

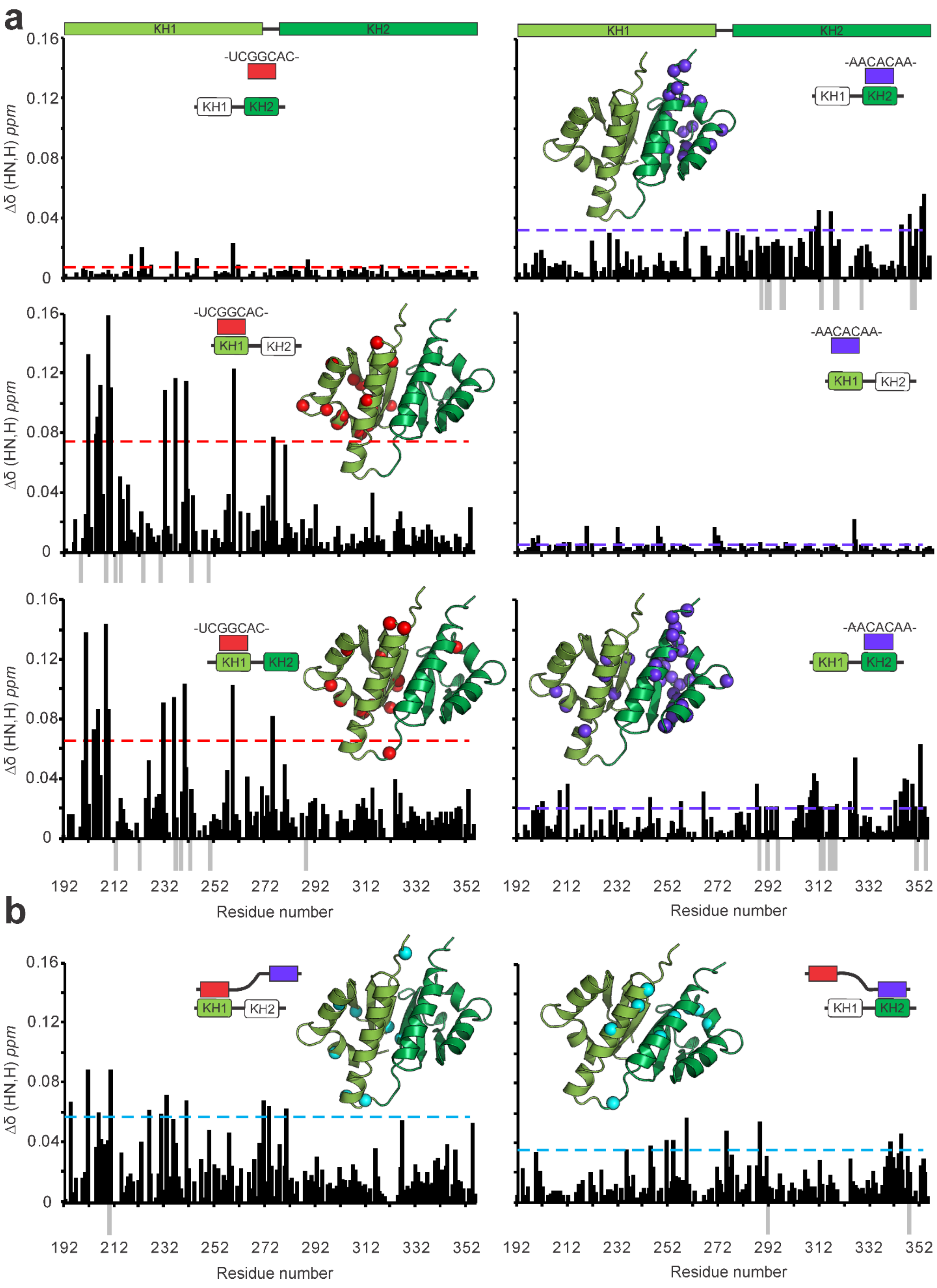

**Supplementary Figure 7.**
**Structure and RNA recognition by the IMP3 tandem KH1-2** (**related to Fig. 4**).

(**a, b**) Overview of all combined CSPs plotted along the KH1-2 primary sequence as derived from spectra in respective panels **a**) and **b**) of **Supplementary Fig. 6**. KH1 and 2 subdomains are indicated on top. Grey bars indicate residues that could not be re-assigned unambiguously because of line broadening, e.g. as the result of exchange after titration with RNA. Gaps indicate unassigned residues or prolines. The dotted line indicates the average plus twofold standard deviation of all shown CSPs within a given experiment and is interpreted as the threshold of significance. The insets show mapping of significantly shifting and line-broadened residues to the KH1-2 crystal structure. The items summarize the proposed mode of interaction underlying the respective titration. Note that icons for RNAs are shown in 3'-5' orientation in this figure.

## Supplementary Figure 8

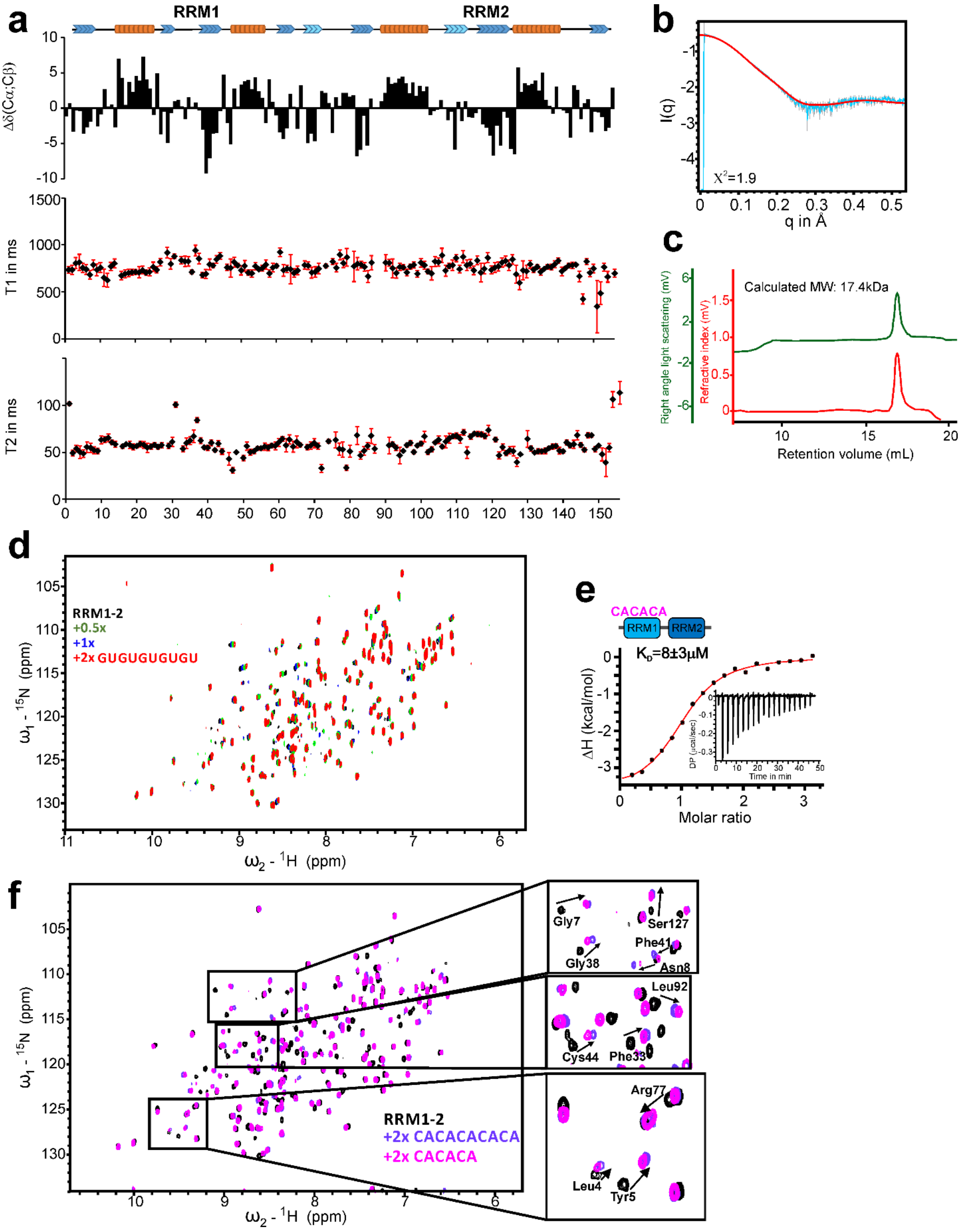

**Supplementary Figure 8.**
**RNA binding by the IMP3 RRM1-2 tandem domains (related to Fig. 5).**

(**a**) Top, scheme of RRM1-2 secondary structure elements as obtained from secondary chemical shifts in the panel below. Blue represents $\beta$-strands, red indicates $\alpha$-helices. The middle and lower panels show the $^{15}$N $T_1$ and $T_2$ relaxation times in RRM1-2. Error bars show the uncertainty derived from fitting relaxation curves of T1 and T2, respectively.
(**b**) Overlay of the SAXS scattering curve measured from RRM1-2 at 7 mg/ml (blue) and a theoretical curve obtained from the model shown in **Fig. 5f** and created with the program *Crysol* (red)[63]. The fit is given in the plot.
(**c**) Static light scattering analysis coupled to analytical size exclusion chromatography of RRM1-2 indicating the protein is a pure monomer as shown by both the right angle light scattering and the refractive index. The derived MW is denoted; the theoretical value is 17.8 kDa.
(**d**) $^{1}$H-$^{15}$N-HSQC overlay of RRM1-2 alone and in complex with different concentrations of $(GU)_5$ RNA (see color code).
(**e**) ITC curve of RRM1-2 when titrated to $(CA)_3$ RNA forming a 1:1 complex. The suggested mode of complex formation and the binding constant are given. Values are mean and standard deviation of three experiments. All ITC measurements are summarized in **Supplementary Table 2**.
(**f**) $^{1}$H-$^{15}$N-HSQC overlay of RRM1-2 alone and in complex with twofold excess of $(CA)_3$ and $(CA)_5$. The three zoom-ins show representative residues affected by RNA binding.
Source data are provided as a Source Data file.

## Supplementary Figure 9

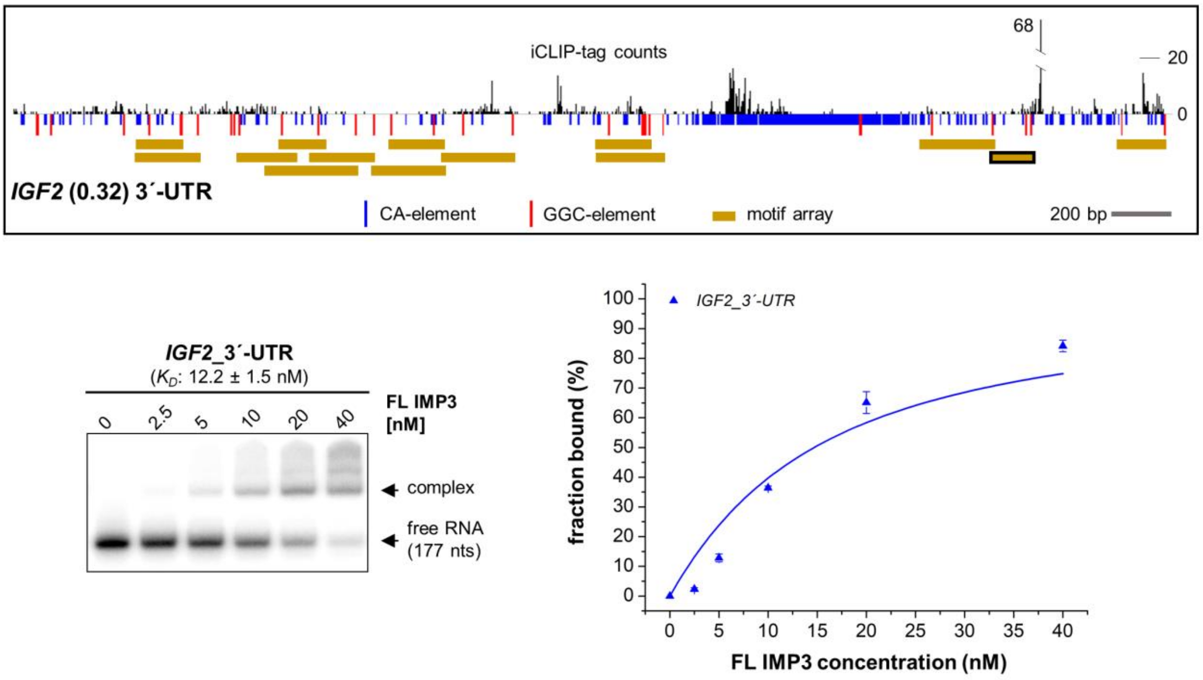


**Supplementary Figure 9.**

**IMP3 binding sites within the 3´-UTR of *IGF2* (related to Fig. 7).**

SELEX motifs within the 3´-UTR of the natural IMP3 target *IGF2* predicted from iCLIP data and validated by EMSA. iCLIP-tags (top, black) and CA/GGC-elements (violet/red, bottom) as well as predicted IMP3-motif arrays (brown bars, bottom) are indicated. An RNA (177 nts; 5 nM and $^{32}$P-labeled) with the region of the highest iCLIP-tag peak (indicated by a black box) was incubated with 0-40 nM full-length (FL) IMP3. The corresponding binding curve for $K_D$-estimation is shown on the right (mean and standard error of three experiments, data fitted with the quadratic binding equation).
Source data are provided as a Source Data file.

**Supplementary Table 1.**

| | KH1-Δ2 (PDB ID 6GQE) | | |
|---|---|---|---|
| **Data collection** | | | |
| Space group | $P2_12_12_1$ | | |
| Cell dimensions | | | |
| *a*, *b*, *c* (Å) | 36.20 | 58.09 | 60.87 |
| α, β, γ (°) | 90.0 | 90.0 | 90.0 |
| Resolution (Å) | 50–2.15 (2.21–2.15) | | |
| $R_{merge}$ | 8.4 (97.3) | | |
| $I / \sigma(I)$ | 11.94 (1.63) | | |
| $CC_{1/2}$ | 99.9 (76.7) | | |
| Completeness (%) | 100 (99.9) | | |
| Redundancy | 6.5 (6.9) | | |
| **Refinement** | | | |
| Resolution (Å) | 2.15 | | |
| No. reflections | 7,366 | | |
| $R_{work}$ / $R_{free}$ | 23.29 / 29.27 | | |
| No. atoms | | | |
| Protein | 1,285 | | |
| Water | 16 | | |
| *B*-factor overall | 39.27 | | |
| R.m.s. deviations | | | |
| Bond lengths (Å) | 0.007 | | |
| Bond angles (°) | 1.25 | | |
| Ramachandran plot | | | |
| Most favored (%) | 99 | | |
| Additional allowed (%) | 1 | | |

*Values in parentheses are for highest-resolution shell.

Data collection and refinement statistics for KH1-Δ2.

## Supplementary Table 2.

| KH1-2 | CGG 7-mer | | | | CA 7-mer | | | | 34-mer | | | |
|---|---|---|---|---|---|---|---|---|---|---|---|---|
| | $K_D$ | ΔH | -TΔS | n | $K_D$ | ΔH | -TΔS | n | $K_D$ | ΔH | -TΔS | n |
| **WT** | 45±8 | -14.8±5 | 12.1±5.8 | 1.0±0.1 | >100* | | | | 4±2 | -25.2±3.8 | -10.4±4.3 | 0.85±0.1 |
| **Δ2** | 39±8 | -9.3±3.5 | 7.4±4.1 | 1.0±0.1 | n.b. | | | | n.d. | | | |
| **Δ1** | n.b. | | | | >100* | | | | n.d. | | | |
| **Δ1Δ2** | n.b. | | | | n.b. | | | | n.b. | | | |
| | | | | | | | | | | | | |
| **RRM1-2** | $(CA)_5$ | | | | $(CA)_3$ | | | | $(GU)_5$ | | | |
| | $K_D$ | ΔH | -TΔS | n | $K_D$ | ΔH | -TΔS | n | $K_D$ | ΔH | -TΔS | n |
| **WT** | 9±2 | -9.4±0.9 | 2.7±0.7 | 1.05±0.15 | 8±3 | -5.4±1.1 | -1.6±0.4 | 0.95±0.1 | 49±3 | -2.2±0.4 | -3.7±0.9 | 1.1±0.1 |

n.b., no binding; n.d., not determined, *as estimated from NMR titration

Summary of ITC measurements of IMP3 constructs with RNAs as denoted. Given are average $K_D$ values (μM), thermodynamic parameters ΔH and TΔS (kcal/mol), and stoichiometries (n) plus/minus standard deviations, as obtained from at least three independent experiments. Source data are provided as a Source Data file.
