# Supplementary material for: Combinatorial recognition of clustered RNA elements by the multidomain RNA-binding protein IMP3
Source: Nat Commun. 2019 May 22;10:2266. doi: 10.1038/s41467-019-09769-8 (PMC6531468; doi:10.1038/s41467-019-09769-8)
Supplement: Supplementary file 2 — Reporting Summary [file 41467_2019_9769_MOESM2_ESM.pdf]

nature research

Corresponding author(s): *Double-blind peer review submissions: write DBPR and your manuscript number here instead of author names.*

Reporting Summary

Nature Research wishes to improve the reproducibility of the work that we publish. This form provides structure for consistency and transparency in reporting. For further information on Nature Research policies, see Authors & Referees and the Editorial Policy Checklist.

Statistical parameters

When statistical analyses are reported, confirm that the following items are present in the relevant location (e.g. figure legend, table legend, main text, or Methods section).

n/a	Confirmed	
☐	☒	The exact sample size (n) for each experimental group/condition, given as a discrete number and unit of measurement
☐	☒	An indication of whether measurements were taken from distinct samples or whether the same sample was measured repeatedly
☐	☒	The statistical test(s) used AND whether they are one- or two-sided<br>*Only common tests should be described solely by name; describe more complex techniques in the Methods section.*
☒	☐	A description of all covariates tested
☒	☐	A description of any assumptions or corrections, such as tests of normality and adjustment for multiple comparisons
☒	☐	A full description of the statistics including central tendency (e.g. means) or other basic estimates (e.g. regression coefficient) AND variation (e.g. standard deviation) or associated estimates of uncertainty (e.g. confidence intervals)
☒	☐	For null hypothesis testing, the test statistic (e.g. F, t, r) with confidence intervals, effect sizes, degrees of freedom and P value noted<br>*Give P values as exact values whenever suitable.*
☒	☐	For Bayesian analysis, information on the choice of priors and Markov chain Monte Carlo settings
☒	☐	For hierarchical and complex designs, identification of the appropriate level for tests and full reporting of outcomes
☒	☐	Estimates of effect sizes (e.g. Cohen's d, Pearson's r), indicating how they were calculated
☐	☒	Clearly defined error bars<br>*State explicitly what error bars represent (e.g. SD, SE, CI)*

Our web collection on statistics for biologists may be useful.

Software and code

Policy information about availability of computer code

Data collection	No undescribed software was used.
Data analysis	No undescribed software was used.

For manuscripts utilizing custom algorithms or software that are central to the research but not yet described in published literature, software must be made available to editors/reviewers upon request. We strongly encourage code deposition in a community repository (e.g. GitHub). See the Nature Research guidelines for submitting code & software for further information.

Data

Policy information about availability of data

All manuscripts must include a data availability statement. This statement should provide the following information, where applicable:

- Accession codes, unique identifiers, or web links for publicly available datasets
- A list of figures that have associated raw data
- A description of any restrictions on data availability

KH1-2 (IMP3), X-ray crystallographic structure: Fig. 4, Atomic coordinates and structure factors have been deposited in the Protein Data Bank under the accession code 6GQE.
NMR amide chemical shifts for RRM1-2, KH1-2 wt, KHdelta1-2, and KH1-delta2 are deposited in the BMRB with accession codes 27813, 27815, 27827, and 27186.

IMP3 iCLIP (HepG2 cells): Fig. 7, Sequencing data for the IMP3 iCLIP in HepG2 have been deposited in the Sequence Read Archive (SRA) of NCBI under the accession code SRP139915.

Field-specific reporting

Please select the best fit for your research. If you are not sure, read the appropriate sections before making your selection.

☒ Life sciences ☐ Behavioural & social sciences ☐ Ecological, evolutionary & environmental sciences

For a reference copy of the document with all sections, see nature.com/authors/policies/ReportingSummary-flat.pdf

Life sciences study design

All studies must disclose on these points even when the disclosure is negative.

Sample size	Sample sizes were estimated on the basis of previous studies using similar methods and analyses that are published.
Data exclusions	n/a
Replication	As specified in figure legends, when applicable.
Randomization	Samples were not randomized across experiments.
Blinding	Blinding was not applicable to the experiments done.

Reporting for specific materials, systems and methods

Materials & experimental systems

n/a	Involved in the study	
☒	☐	Unique biological materials
☐	☒	Antibodies
☐	☒	Eukaryotic cell lines
☒	☐	Palaeontology
☒	☐	Animals and other organisms
☒	☐	Human research participants

Methods

n/a	Involved in the study	
☒	☐	ChIP-seq
☒	☐	Flow cytometry
☒	☐	MRI-based neuroimaging

Antibodies

Antibodies used	IMP1 (BSBS AB facility, clone 6A9, used in Fig. 8b)<br>IMP2 (BSBS AB facility, clone 6A12, used in Fig. 8b)<br>IMP3 (Millipore, Cat.No.: 07-104, used in Fig. 3c, 7c and 8b)<br>IMP3 (BSBS AB facility, clone 6G8, used for validation of ES-2 IMP3 KO cell lines)<br>IMP3 (MBL, RN009P, used for validation of ES-2 IMP3 KO cell lines)<br>GAPDH (Sigma, Cat.No.: G8795, used in Fig. 8b)<br>GST (Pharmacia Biotech, Cat.No.: 27-4577-01, used in Fig. 3c)<br>FLAG (Sigma, Cat.No.: F3165, used in Fig. 7c)
Validation	All commercial antibodies are reported by the manufacturers as suitable for the applied method (e.g. Western blotting).<br>All non-commerical antibodies were validated by the corresponding facility.

Eukaryotic cell lines

Policy information about cell lines

Cell line source(s)	HeLa cells: commercially obtained from the DSMZ (German Collection of Microorganisms and Cell Cultures).<br>ES-2 cells: WT and IMP3-KO cell lines were provided by the Hüttelmaier lab (initially obtained from the ATCC, American Type Culture Collection).
Authentication	HeLa cells were authenticated by the DSMZ.<br>ES-2 cell derivatives were generated and authenticated by the Hüttelmaier lab.

Mycoplasma contamination	Cell culture was routinely tested for Mycoplasma contamination. The results were negative.
Commonly misidentified lines<br>(See ICLAC register)	No commonly misidentified cell lines were used.
