# Supplementary material for: Combinatorial recognition of clustered RNA elements by the multidomain RNA-binding protein IMP3
Source: Nat Commun. 2019 May 22;10:2266. doi: 10.1038/s41467-019-09769-8 (PMC6531468; doi:10.1038/s41467-019-09769-8)
Supplement: Supplementary file 3 — Description of Additional Supplementary Information [file 41467_2019_9769_MOESM3_ESM.pdf]

Description of Additional Supplementary Files

File Name: Supplementary Data 1
Description: SELEX-seq motif enrichment analysis

File Name: Supplementary Data 2
Description: 4-mer motif combinations used for spacing analysis

File Name: Supplementary Data 3
Description: List of oligonucleotides
